# A (–)-kolavenyl diphosphate synthase catalyzes the first step of salvinorin A biosynthesis in *Salvia divinorum*

**DOI:** 10.1093/jxb/erw493

**Published:** 2017-02-15

**Authors:** Xiaoyue Chen, Anna Berim, Franck E. Dayan, David R. Gang

**Affiliations:** 1 Institute of Biological Chemistry, Washington State University, Pullman, WA 99164,USA; 2 Bioagricultural Sciences and Pest Management, Colorado State University, Fort Collins, CO 80523–1177, USA

**Keywords:** class II diterpene synthase, diterpenoid diversification, (–)-kolavenyl diphosphate, (–)-kolavenol, neo-functionalization, neo-clerodane diterpenoid, product specificity, repeated evolution, *Salvia divinorum*, salvinorin A biosynthesis

## Abstract

*Salvia divinorum* (Lamiaceae) is an annual herb used by indigenous cultures of Mexico for medicinal and ritual purposes. The biosynthesis of salvinorin A, its major bioactive neo-clerodane diterpenoid, remains virtually unknown. This investigation aimed to identify the enzyme that catalyzes the first reaction of salvinorin A biosynthesis, the formation of (–)-kolavenyl diphosphate [(–)-KPP], which is subsequently dephosphorylated to afford (–)-kolavenol. Peltate glandular trichomes were identified as the major and perhaps exclusive site of salvinorin accumulation in *S. divinorum*. The trichome-specific transcriptome was used to identify candidate diterpene synthases (diTPSs). *In vitro* and *in planta* characterization of a class II diTPS designated as SdKPS confirmed its activity as (–)-KPP synthase and its involvement in salvinorin A biosynthesis. Mutation of a phenylalanine into histidine in the active site of SdKPS completely converts the product from (–)-KPP into *ent*-copalyl diphosphate. Structural elements were identified that mediate the natural formation of the neo-clerodane backbone by this enzyme and suggest how SdKPS and other diTPSs may have evolved from *ent*-copalyl diphosphate synthase.

## Highlight

The class II diterpene synthase that catalyzes the first committed reaction of the virtually unknown biosynthetic pathway of salvinorin A in *Salvia divinorum* is identified and the structural basis for product specificity of this enzyme is elucidated.

## Introduction

*Salvia divinorum* (Lamiaceae) is a powerful hallucinogenic plant traditionally used in psycho-spiritual and healing ceremonies by the Mazatecs of Oaxaca in southern Mexico (Wasson, 1962). Its psychoactivity is largely due to salvinorin A, a highly selective kappa-opioid receptor agonist (Roth *et al.*, 2002; Butelman and Kreek, 2015), whose unique structure and distinct pharmacological features make it a valuable template/lead for structure–function explorations (Tejeda *et al.*, 2012; Butelman and Kreek, 2015; Simonson *et al.*, 2015). Other related metabolites from *S. divinorum* such as (–)-kolavenol, hardwickiic acid, and salvinorin B also exhibit strong biological activities (Pittaluga *et al.*, 2013; Marchant *et al.*, 2016). *Salvia divinorum* is the only known natural source of salvinorin A. While total chemical synthesis of salvinorin A is possible (Nozawa *et al.*, 2008), it is not commercially feasible. Elucidation of the natural biosynthetic pathways leading to salvinorin A will facilitate its production and structural diversification for research and medical applications through synthetic biology approaches.

**Fig. 1.**
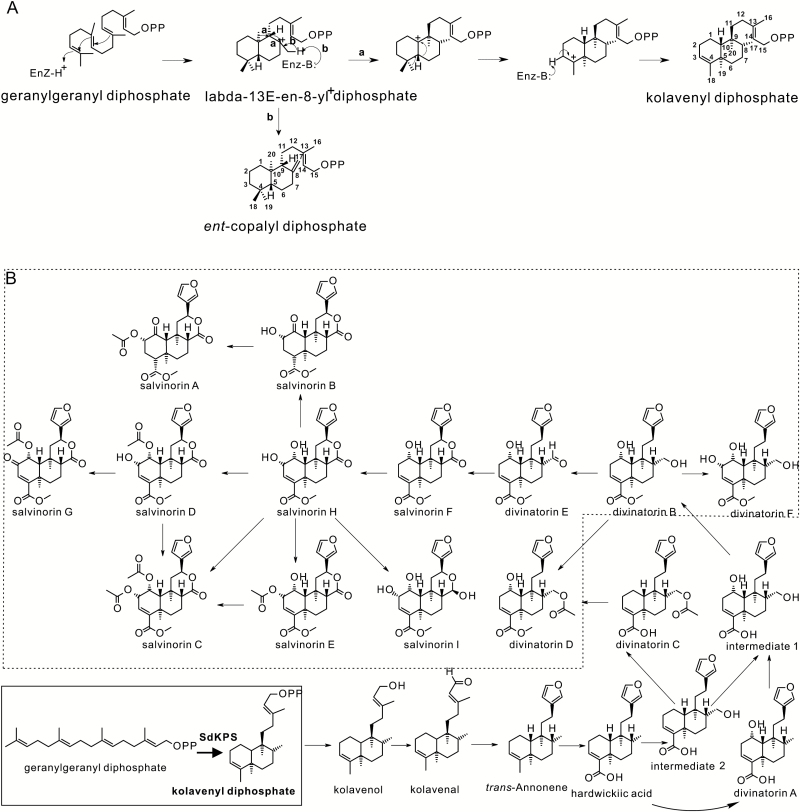
Relevant reactions and proposed salvinorin A biosynthesis pathway. (A) The scheme of reactions catalyzed in routes a and b using GGPP as the substrate with formation of intermediate labda-13-en-8-yl^+^ diphosphate. (B) The proposed biosynthetic pathway of salvinorin A based on known compounds identified from *S. divinorum*. The step elucidated in this study is highlighted in the box to the lower left. The compounds outlined in the dashed box could exist as the corresponding descarboxylmethylated molecules and then be converted to their methylesters just prior to formation and secretion. Alternatively, the carboxylmethyltransferase could act early in the pathway.

Structurally, salvinorin A is a neo-clerodane diterpenoid, belonging to the superfamily of labdane-related diterpenoids, which currently accounts for over 7000 distinct chemical entities (Peters, 2010; Zi *et al.*, 2014). Further subdivision of clerodane diterpenoids into *neo*- and *ent-neo*-clerodanes is based on the stereochemical configurations of their hydrocarbon backbones (Tokoroyama, 2000). The biosynthesis of diterpenoids in plants begins in plastids with the formation of their common precursor, geranylgeranyl diphosphate (GGPP) via the deoxyxylulose phosphate pathway. In angiosperms, labdane-related diterpenoids are then typically produced in two reaction steps by pairs of often plastidial class II and class I diterpene synthases (diTPSs) (Peters, 2010). The first step, the protonation-initiated cyclization of GGPP, is mediated by a class II diTPS through formation of the common bicyclized labda-13-en-8-yl^+^ diphosphate intermediate (Fig. 1A) and its conversion into bicyclic prenyl diphosphates (Peters, 2010). The structural diversity of hydrocarbon skeletons generated by class II-catalyzed cyclization reactions largely depends on the absolute configuration of this intermediate and how it is further processed. For instance, deprotonation of the methyl group at C-8 of the intermediate leads to formation of copalyl diphosphate (CPP) isomers, with *ent*-CPP (9*R*, 10*R*, Fig. 1A) and ‘normal’ CPP (9*S*, 10*S*) being the most frequently observed reaction products (Xu *et al.*, 2007*a*; Keeling *et al.*, 2010; Brückner *et al.*, 2014; Pateraki *et al.*, 2014; Cui *et al.*, 2015). If the carbocation is captured by water prior to deprotonation, labda-13-en-8-ol diphosphate is formed instead (Schalk *et al.*, 2012; Zerbe *et al.*, 2013; Pateraki *et al.*, 2014; Cui *et al.*, 2015; Andersen-Ranberg *et al.*, 2016). Notably, labda-13-en-8-yl^+^ diphosphate can also be rearranged through a series of 1,2-hydride and methyl shifts to give rise to a diphosphate with the clerodane backbone (Fig. 1A, route a) (Peters, 2010). Products of the class II diTPSs are substrates for class I diTPSs, which cleave the diphosphate group, usually followed by cationic cyclization and/or rearrangement reactions. The biosynthesis of the diverse labdane-related diterpenoids in higher plants is thought to have evolved from ancestral gibberellin biosynthesis, which involves cyclization of GGPP to *ent*-CPP by a class II diTPS, *ent*-copalyl diphosphate synthase (CPS), followed by the formation of *ent*-kaurene by a class I diTPS (Zi *et al.*, 2014).

Based on the structures of diterpenoids isolated from *S. divinorum* (Valdes *et al.*, 1984; Valdés *et al.*, 2001; Bigham *et al.*, 2003; Munro and Rizzacasa, 2003; Kutrzeba *et al.*, 2009*a*), we have outlined a proposed biosynthetic route to salvinorin A (Kutrzeba *et al.*, 2007, 2009*b*) (Fig. 1B). The backbone of salvinorin A and the common modular mechanism employed in the biosynthesis of labdane-related diterpenoids allowed us to hypothesize that salvinorin A biosynthesis involves a class II diTPS catalyzing the conversion of GGPP into (–)-kolavenyl diphosphate [(–)-KPP], which is further dephosphorylated by a class I diTPS into (–)-kolavenol, the first isolated putative pathway intermediate. Subsequent modifications yield salvinorin A and are accompanied by the accumulation of an array of related diterpenoids representing pathway intermediates and minor products (Fig. 1B). This current investigation identified and characterized the (–)-KPP synthase (SdKPS) involved in salvinorin A biosynthesis.

## Materials and Methods

### Chemical sources

GGPP was obtained from Echelon (Salt Lake City, UT, USA). (–)-Kolavenol and hardwickiic acid were purchased from BioBioPha (Kunming, Yunnan, China). Salvinorin B and salvinorin A were from Sigma-Aldrich. Apigenin was from Indofine.

### Plant material growth and culture

*S. divinorum* (line ‘Tucson’) plants were clonally propagated from stem cuttings placed in glass flasks filled with distilled water and transplanted to soil (Frog Smart Naturals potting soil, Foxfarm Soil & Fertilizer Co., Arcata, CA, USA) after 4 weeks of root formation. Plants (during and after rooting) were grown under controlled conditions with a 16/8 h light/dark cycle, temperatures of 24 /18 °C, and humidity kept at 20–50% in a growth room. Fluorescent lamps (Spectralux) produced light intensities between 100 PAR (plant bases) and 450 PAR (plant tops).

### Metabolite extracts

*Salvia divinorum* tissue samples and leaf disc samples of *Nicotiana benthamiana* were ground under liquid nitrogen with a mortar and pestle. A portion of frozen powder was used for RNA extraction for transcript profiling of *S. divinorum* leaves at different stages. Around 200–250 mg of frozen powder was weighed and suspended in 2 ml of a methyl tert-butyl ether (MTBE):ethyl acetate (2:1, v/v) mixture for metabolite analysis. After extraction overnight at room temperature under shaking, the suspension was centrifuged at 21,000 *g* for 10 min, the supernatant was carefully removed and stored at –20 °C, and the remaining tissue was extracted overnight with 1 ml of solvent mix as above. Combined extracts were dried under a stream of nitrogen, and the dry residue was dissolved in 500 µl of ethyl acetate. An aliquot of 200 µl was again dried under nitrogen, the residue was dissolved in 100 µl of methanol, and 2–5 µl of this solution was injected for liquid chromatography–mass spectrometry (LC-MS) analysis.

Young leaves (~2 cm in length) were chosen for peltate glandular trichome secretory cell cluster (gland) isolation either for metabolite extraction or RNA isolation (Gang *et al.*, 2001), and a total of 8 g of fresh young leaves were used per isolation batch. The *S. divinorum* peltate glands were washed and collected from a 33-µm mesh cloth, yielding about 50 µl of glands per batch. Isolated glands were disrupted by sonication (three 10-s pulses in a Branson 450 sonifer with a microtip; Branson Ultrasonics) and 1-ml MTBE:ethyl acetate (2:1, v/v) mixture was added immediately after sonication. The procedure for metabolite extraction was the same as above.

### Metabolite and enzyme assay analyses

Metabolites and products of all enzymatic assays were analyzed using an LC-MS system (Acquity ultra-performance liquid chromatography coupled to a Synapt G2-S HDMS quadrupole-ion mobility spectrometry–time of flight mass spectrometer; Waters). Extracts from *Salvia* tissues were separated on an Acquity UPLC BEH C_18_ column (50 × 2.1 mm, 1.7 µm; Waters) at a flow rate of 0.45 ml min^–1^ using a linear gradient of acetonitrile (B) and water (A) with 0.1% (v/v) formic acid: 0 min, 7% B; 8 min, 99% B; 10 min, 99% B; 10.1 min, 7% B; 13 min, 7% B. Dephosphorylated enzymatic products were separated on a CORTECS UPLC C18^+^ column (100 × 2.1 mm, 1.6 µm; Waters) using the same gradient and flow rate. Positive-mode electrospray ionization (ESI) was applied for ionization. The Q-TOF-MS instrument parameters were: source 3.2 kV at 100 °C; desolvation temperature of 250 °C; desolvation gas flow of 800 l h^–1^. The transfer collision energy for MS^e^ and MS/MS fragmentation was 15 eV. Standard curves of serial dilutions of authentic standards were used for quantification. The direct SdKPS enzyme product and GGPP were separated on an Acquity UPLC BEH C_18_ column (50 × 2.1 mm, 1.7 µm; Waters) with 10 mM ammonium bicarbonate buffer (A) and acetonitrile (B) as the mobile phase using the same linear gradient and flow rate as above, and were ionized under negative ESI mode.

### Cloning and heterologous expression of full-length and truncated SdKPS and its mutants

The open reading frames of full-length (Fl) and truncated (Tr) SdKPS were obtained with primers that amplified cDNA from the targeted sites (SdKPS-FULL-F and SdKPS-exp-R for Fl-SdKPS, SdKPS-truncation-F and SdKPS-exp-R for Tr-SdKPS; for details see Supplementary Table S1 at *JXB* online), followed by transfer into the pCR2.1 TOPO TA cloning vector (Invitrogen), and verification by complete sequencing. The resulting clones were subsequently transferred to the pEXP-5-CT/TOPO vector by PCR, giving rise to pEXP/Fl-SdKPS and pEXP/Tr-SdKPS vectors, followed by sequence verification. Site-directed mutagenesis of Tr-SdKPS was performed using the QuikChange Lightning kit (Agilent) with the pEXP/Tr-SdKPS vector. The resulting mutant genes were verified by complete sequencing. The truncated coding sequences of SdKSL1-SdKSL3 were cloned into the pCR2.1 TOPO TA vector with restriction enzyme sites, and digested with corresponding restriction enzymes. SdKSL1 and SdKSL3 were subcloned into the pET15b vector (Merck), and SdKSL2 was subcloned into the pCOLADuet^TM^-1 (Novagen) expression plasmid. The expression of recombinant proteins was carried out in BL21 Star^TM^ (DE3) (ThermoFisher) cells induced via the auto-induction method (Studier, 2005) at 18 °C to 20 °C. Pelleted cells were suspended in binding buffer (20 mM HEPES, pH 7.5, 500 mM NaCl, 5 mM imidazole, and 5% glycerol) containing 0.01% (w/v) lysozyme and then disrupted by sonication. The supernatant after centrifugation (20 000 *g*, 4 °C, 10 min) was loaded to a pre-equilibrated nickel-nitrilotriacetic acid agarose matrix (Qiagen). The slurry was incubated with mild agitation at 4 °C for 1 h and then was washed with 10 volumes of washing buffer (20 mM imidazole). The purified protein was eluted from the resin with buffer containing 350 mM imidazole and then transferred into storage buffer (50 mM HEPES, pH 7.5, 1 mM dithiothreitol, and 10% glycerol), and stored at –80 °C if not used immediately.

### Enzyme activity characterization

The reaction conditions were optimized in order to achieve reaction velocity proportional to reaction time and protein amount. Kinetic parameters were derived from fitting the observed data to the Michaelis–Menten model (GraphPad Prism 6). The pH optimum was determined over a pH range from 5.8 to 8.2 in 0.2 unit increments in 50 mM of Bis-Tris buffer and Bis-Tris-Propane buffer. The concentration of Mg^2+^ was compared at 0.01 mM, 0.1 mM, 1 mM, and 10 mM. TES, Bis-Tris, HEPES, and Tris at pH 7.0 were compared as buffer systems. For kinetics measurements, SdKPS enzyme assays were performed at 30 °C in 250 µl of assay buffer (50 mM HEPES, pH 7.0, 100 mM KCl, 0.1 mM MgCl_2_, 10% glycerol). SdKPS activity was characterized by assaying 0.5 µg purified recombinant protein incubated with 1–25 µM GGPP in assay buffer for 30 s. Reactions were terminated as described previously by Prisic and Peters (2007): a volume of 50 µl 20 mM N-ethylmaleimide (NEM) in 500 mM Gly, pH 11 was added, followed by incubation at 75 °C for 5 min. Excess NEM was deactivated by adding 20 µl of 1 M DTT and incubating for 15 min at room temperature, then neutralizing with 25 µl of 1 M HCl. Ten units of calf intestine phosphatase (NEB) were added to dephosphorylate both substrate and product for 30 min at 37 °C. Catalytic turnover was determined from the ratio of product to the sum of product and substrate and the known starting GGPP concentration. Kinetic parameters were calculated from three independent assay series, each with two technical replicates. Control assays included empty vector controls, no-substrate assays, and assays terminated by adding 50 µl of 20 mM NEM and incubation at 75 °C for 5 min before initiating reaction.

### T-DNA construction and transient expression of SdKPS in *N. benthamiana*

*Agrobacterium tumefaciens* strain GV3101:pMP90 was transformed with full-length SdKPS coding sequence in the T-DNA vector pEarleygate 100 (Earley *et al.*, 2006) by electroporation. The *Agrobacterium* infiltration was adapted from previous methods (Voinnet *et al.*, 2003; Sparkes *et al.*, 2006). Transformed single colonies grown on Luria-Bertani (LB) agar plates containing kanamycin (50 µg ml^–1^) and rifampicin (25 µg ml^–1^) were used to inoculate 2 ml LB medium plus appropriate antibiotics at 28 °C and 180 rpm for about 24 h. The pelleted cells (1000 *g*, 24 °C, 10 min) were washed with sterile water before being re-suspended in infiltration medium (27 mM glucose, 50 mM MES, 10 mM MgCl_2_, 0.1 mM acetosyringone). When several constructs were co-infiltrated, the corresponding *Agrobacterium* cells were mixed together in equal proportion. The mixture was infiltrated into the leaves of 4-week-old *N. benthamiana* plants. The plants were kept under normal growth conditions for 4 d until metabolite extraction. *A. tumefaciens* strain GV3101:pMP90 carrying a T-DNA expressing the silencing suppression p19 protein was mixed together with the construct of interest to suppress RNA silencing.

### Total RNA extraction

For cloning and Illumina cDNA library construction, isolated glandular trichomes were disrupted by sonication as described above and RNA was purified using the RNeasy Plant Mini Kit (Qiagen). Total RNA for quantitative real-time PCR was extracted from ground leaves of different developmental stages using the same kit. Genomic DNA was removed using the TURBO DNase kit (Ambion). Final RNA concentration was determined using a Nanodrop 2000 (Thermo).

### Quantitative real-time PCR

The relative quantification method (Schmittgen and Livak, 2008) was performed to measure the abundance of SdKPS transcripts. After DNase treatment, 300 ng total RNA was reverse transcribed into cDNA using qScript™ cDNA Supermix kit (Quanta BioSciences). Elongation factor-1 (EF-1) was tested with cDNAs from leaves of different developmental stages and was chosen as the reference gene for normalization (see Supplementary Table S2). Primer sequences are shown in Supplementary Table S1 (SdKPS-F and SdKPS-R, EF-1-F and EF-1-R). Annealing temperatures between 53 °C and 59.5 °C were compared, and 55 °C was suited for both SdKPS and SdEF-1. Amplification efficiencies of both genes were compared using five serial cDNA dilutions. PerfeCta® SYBR® Green FastMix® (Quanta BioSciences) was used for the PCR reaction of 95 °C for 2 min, 40 cycles of 95 °C for 10 s, 55 °C for 15 s, and 70 °C for 20 s, with a subsequent melting curve from 60 °C to 95 °C over 20 min (in a realplex2 Mastercycler EP gradient S, Eppendorf). cDNA corresponding to 25 ng total RNA was used as template. Expression levels of SdKPS were normalized to those of SdEF-1 using the comparative C_t_ method. Five biological replicates of each leaf pair were analyzed with three technical replicates.

### Illumina sequencing and assembly

Total RNAs from peltate trichomes were used for constructing Illumina paired-end cDNA libraries, sequencing, and read assembly as described previously by He *et al.* (2012).

### Sequence similarity analysis of *S. divinorum* diTPSs

Protein sequences were aligned using ClustalX2 (Larkin *et al.*, 2007). The neighbor-joining algorithm (Saitou and Nei, 1987) was used with 1000 bootstrap replicates (Felsenstein, 2010) in MEGA6 (Tamura *et al.*, 2013) to generate similarity relationships of candidate *S. divinorum* diTPSs and other diTPSs. The *ent*-kaurene/kaurenol synthase from *Physcomitrella patens* was used to root the tree. Accession numbers of included proteins are given in Supplementary Table S3.

### Protein structure modeling

Homology models were built using I-TASSER (Zhang, 2008; Roy *et al.*, 2010) (http://zhanglab.ccmb.med.umich.edu/I-TASSER/). The model with the highest c-score was selected for structural studies. Substrate analog AG8 {S-[(2E,6E,10E)-14-(dimethylamino)-3,7,11-trimethyltetradeca-2,6,10-trien-1-yl] trihydrogen thiodiphosphate} was docked with each model as the ligand. The 3D structure of the labda-13*E*-en-8-yl^+^ diphosphate ligand was generated by CORINA classic (Sadowski *et al.*, 1994) (http://www.molecular-networks.com). Molecular docking of the ligand into active sites was performed by AutoDock Vina (Steffen *et al.*, 2010). Models and docking results were viewed and manipulated using PYMOL (DeLano Scientific, South San Francisco, CA, USA).

### MALDI-FTICR-MS imaging

Fresh leaves of *S. divinorum* were affixed to a glass slide using double-sided tape (3M) and 2,5-dihydroxy benzoic acid (matrix compound) was applied at a rate of 0.8 mg min^–1^ (total of 1.07 mg cm^–2^ applied) from a solution of 10 mg ml^–1^ (in methanol–water, 1:1, v/v) by a robotic TM-sprayer (HTX Technologies, Carrboro, NC). MALDI-MS imaging was carried out using a MALDI solariX 9.4T FTICR mass spectrometer (Bruker Daltonics). The acquisition mass range was set from 100 to 2000 *m*/*z* (mass to charge ratio) with data obtained in the positive ion mode at 2 Hz and mass resolution of 66 000 (at *m*/*z* 400). The data were obtained at 20 μm spatial resolution. The raw data were then processed and ion maps visualized in flexImaging 4.1 (Bruker Daltonics).

### Accession numbers

Nucleotide sequences of enzymes reported in this study have been deposited in GenBank with the following accession numbers: SdKPS, KX268505; SdCPSL1, KX268506; SdCPSL2, KX984339; SdCPSL3, KX984340; SdCPSL4, KX984341; SdKSL1, KX268507; SdKSL2, KX268508; SdKSL3, KX268509 and SdEF-1, KX268510.

## Results

### (–)-Kolavenol and salvinorin A accumulate in peltate glands of *S. divinorum*

**Fig. 2.**
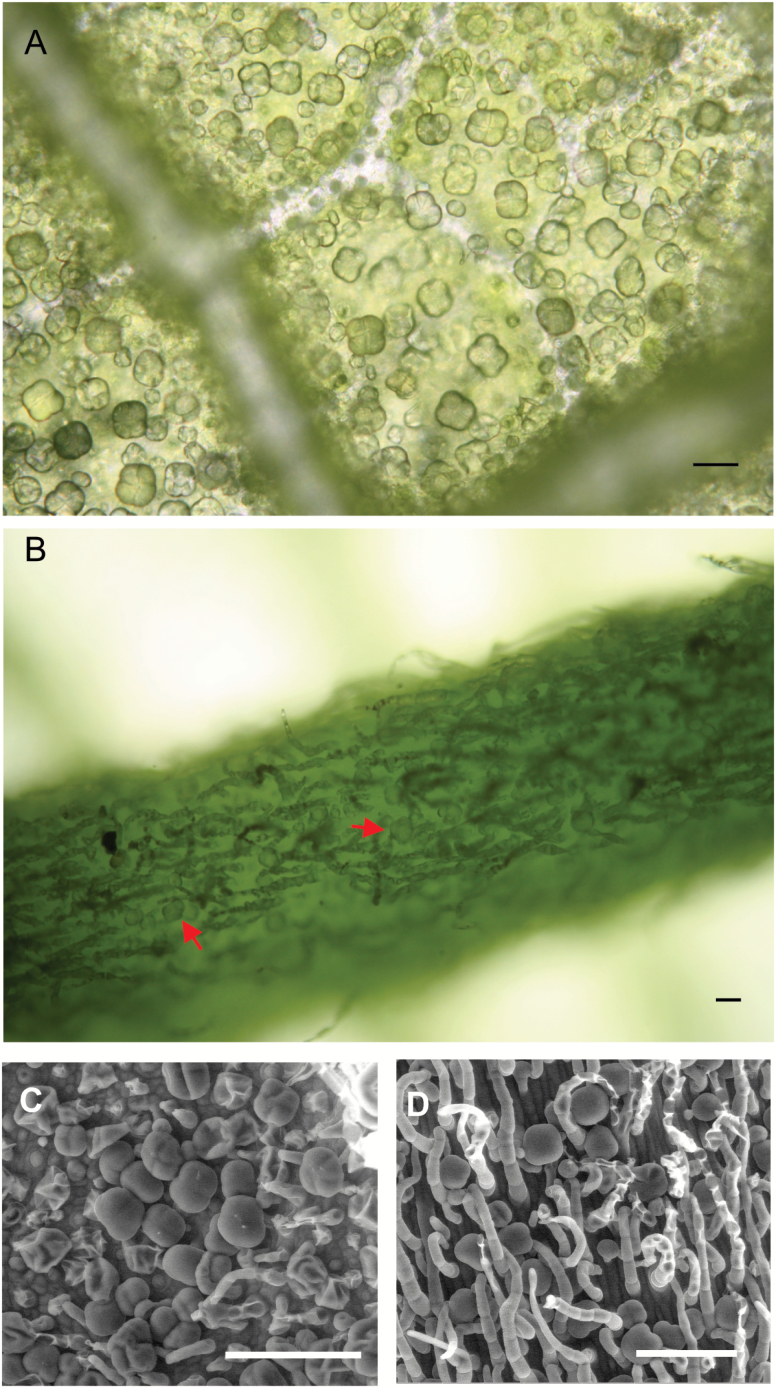
Micrographs of peltate trichomes on the abaxial surface of young leaves of *S. divinorum* (2–3 cm in length) taken with a light microscope (A, B) or by SEM (C, D). (A, C) Leaf surface between veins; (B, D) the central vein. The scale bars are 50 µm in (A, B) and 100 µM in (C, D).

Our first step in understanding the biosynthesis of salvinorin A and related diterpenes focused on identifying the tissues and cellular localization where those diterpenes accumulated. A previous study indicated that salvinorins are stored in the subcuticular space of peltate glandular trichomes of *S. divinorum*, which are located on the abaxial surface of leaves and stems (Siebert, 2004). They are also present on veins (Fig. 2). We then analyzed diterpenoids in extracts from young leaves, stems, and roots. The latter have not been previously tested for the presence of salvinorin diterpenes, even though the roots of some *Salvia* species produce copious amounts of diterpenes (Michavila *et al.*, 1986; Simoes *et al.*, 1986; Ulubelen *et al.*, 1988; Kolak *et al.*, 2009). (–)-Kolavenol, hardwickiic acid, and salvinorin A accumulated at comparable levels in leaves and stems, where peltate trichomes are located (Table 1); however, none of these diterpenoids was detected in extracts from roots, which lack peltate trichomes. These findings suggest that (–)-kolavenol, hardwickiic acid, and salvinorin A might be produced and/or stored in peltate glands.

**Table 1.** *Accumulation of selected diterpenoids in different tissues of* S. divinorum

Tissue	Concentration (µg g^–1^ fresh weight)		
	(–)-Kolavenol	Hardwickiic acid	Salvinorin A
Leaf	0.23 ± 0.02	1.7 ± 0.17	27 ± 1.6
Stem	0.21 ± 0.04	1.0 ± 0.18	46 ± 11
Root	–	–	–

Results are means of four biological replicates ±SE. –, Not detectable.

Using glass bead abrasion (Gang *et al.*, 2001), peltate glands were collected from leaves to investigate the diterpenoid content in trichomes directly. As expected, (–)-kolavenol, hardwickiic acid, and salvinorins A and B were readily detected in the isolated glands. The significantly higher concentrations of the diterpenoids in trichomes compared to those of whole leaves (Table 2) imply that they are strongly enriched in peltate trichomes, as has been observed for other trichome-specific compounds (Berim *et al.*, 2012; Lange and Turner, 2013).

**Table 2.** *Accumulation of selected diterpenoids in whole leaves and peltate glandular trichomes of* S. divinorum. *Results are means of four biological replicates ±SE*

Tissue	Concentration (µg g^–1^ fresh weight of tissue)			
	(–)-Kolavenol	Hardwickiic acid	Salvinorin B	Salvinorin A
Leaf	0.200 ± 0.031	2.00 ± 0.27	31.0 ± 1.6	30.0 ± 2.3
Gland	1100 ± 170	15 000 ± 1100	150 000 ± 24 000	250 000 ± 13 000

**Fig. 3.**
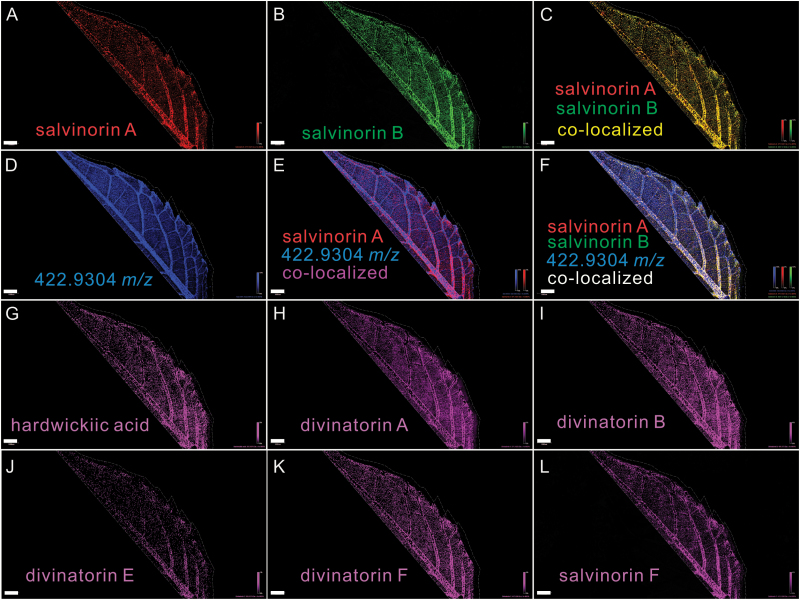
MALDI-FTICR-MS imaging of diterpenoids (including salvinorin A and salvinorin B) on the abaxial surface of a *S. divinorum* leaf. The data were obtained at 20 μm spatial resolution and a mass resolution of >58 000 for all compounds measured (66 000 at *m*/*z* 400), all from the same leaf. Scale bars = 1 mm. Each panel displays the distribution of the mass signal for a specific compound or pairs of compounds using a ±0.1 ppm mass window (achievable using the Bruker MALDI-SolariX 9.4T FTICR instrument) around the monoisotopic mass for each compound. (A) Salvinorin A ([M+K]^+^, *m*/*z* 471.1421) and (B) salvinorin B ([M+K]^+^, *m*/*z* 429.1316). (C) The merged image of (A) and (B). (D) Distribution of an abundant, non-salvinorin-related compound (*m*/*z* 422.9304). (E) The merged image of (A) and (D). (F) The merged image of (A), (B), and (D). (G) Hardwickiic acid ([M+K]^+^, *m*/*z* 355.1676), (H) divinatorin A ([M+K]^+^, *m*/*z* 371.1625), (I) divinatorin B ([M+K]^+^, *m*/*z* 401.1730), (J) divinatorin E ([M+K]^+^, *m*/*z* 399.1574), (K) divinatorin F ([M+K]^+^, *m*/*z* 417.1680) and (L) salvinorin F ([M+K]^+^, *m*/*z* 413.1367).

In an orthogonal direct approach, *in situ* distribution of salvinorin A-related diterpenoids on the abaxial surface of young leaves of *S. divinorum* (2–3 cm in length) was investigated using MALDI-based imaging mass spectrometry (MALDI-IMS). Signals corresponding to salvinorin A (471.1421 *m*/*z*, [M+K]^+^) and salvinorin B (429.1316 *m*/*z*, [M+K]^+^), viewed with a mass window of ±0.1 ppm, appeared as discrete spots distributed on veins and the leaf blade surface (Fig. 3A, B), which is in line with the distribution of *S. divinorum* peltate trichomes (Fig. 2). In addition, these mass signals overlap almost perfectly (Fig. 3C). The co-localization pattern of salvinorin A and B is consistent with the fact that salvinorin B is the proposed immediate precursor of salvinorin A (Fig. 1B). In contrast, numerous non-salvinorin pathway-related compounds showed uniform distribution over the leaf surface (e.g. a randomly selected signal with *m*/*z* 422.9304 is displayed in Fig. 3D) and did not co-localize with salvinorins A and B (Fig. 3E, F). This suggested that salvinorins A and B are only localized to specific sites on the leaf surface (glandular trichomes) and that the other compound was not present in those trichomes. Other intermediates of salvinorin A biosynthesis (salvinorins, divinatorins, hardwickiic acid, etc.) were also detected and largely co-localized with salvinorins A and B (Fig. 3).

### Isolation of a candidate diTPS from *S. divinorum* trichome transcriptome assembly

**Fig. 4.**
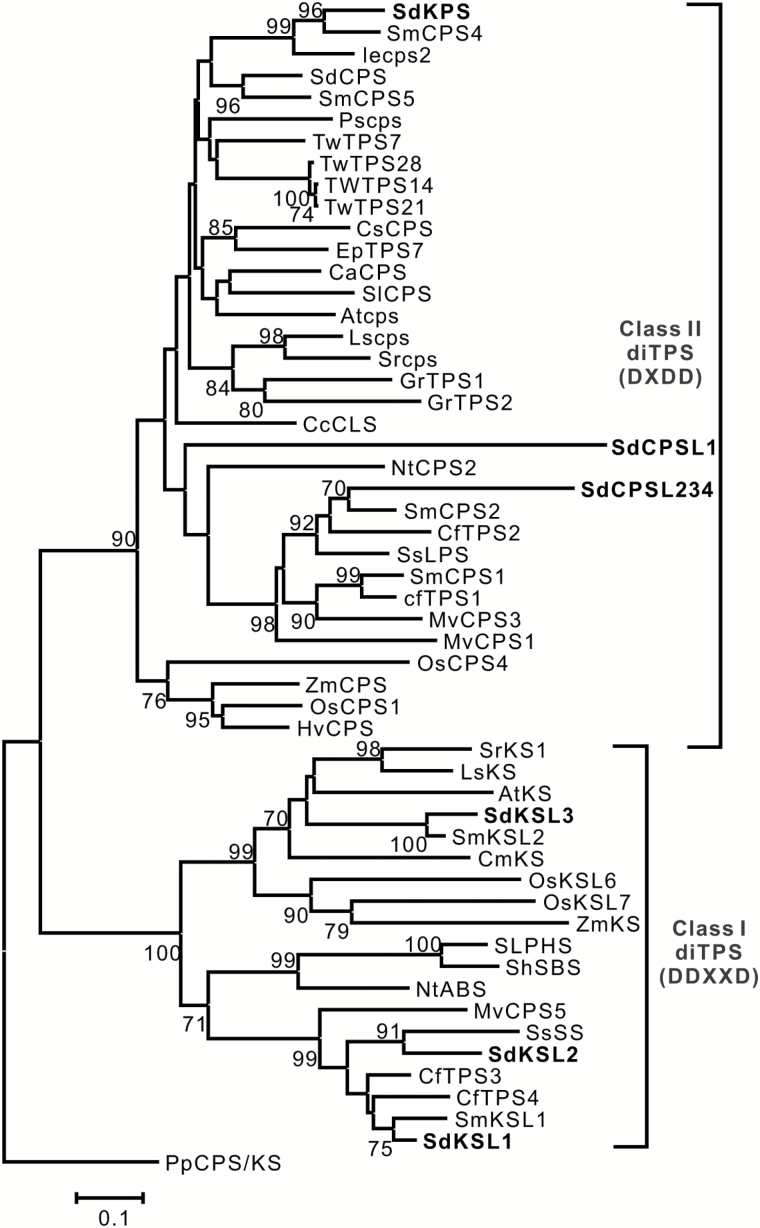
The phylogenetic relationship of *S. divinorum* diTPSs and other published class I and class II diTPSs, as inferred using the Neighbor-Joining method. Bootstrap values (1000 replicates, shown if >70%) are given next to the branches. The scale represents the number of amino acid substitutions per site. The *ent*-kaurene/kaurenol synthase from *Physcomitrella patens* was used to root the tree. For the convenience of phylogeny analysis, we generated the putative chimeric protein sequence, named as SdCPSL234, by combining the translated sequences of SdCPSL2, SdCPSL3, and SdCPSL4 (see Supplementary Fig. S1), as they are likely to be the same gene (Supplementary Tables S1 and S2). Descriptions of proteins are provided in the Materials and Methods and Supplementary Table S4.

Candidate diTPS genes for involvement in salvinorin A biosynthesis were identified by searching our transcriptome assembly, generated using RNA from isolated *S. divinorum* trichomes, using CPS from *Arabidopsis thaliana* (AtCPS, accession number Q38802) as a BLAST query. The best-match sequence was subsequently used as the query for additional BLAST searches against the transcriptome assembly. This exhaustive search revealed a small family of eight putative diterpene synthases belonging to either class I or class II as defined above. Four of the sequences in the database represented full-length transcripts. Each of these was also used to further search the database, but no additional related diTPSs were found. Among the eight genes, a full-length cDNA named SdKPS (Fig. 4) stood out as having the highest read number per length unit (see Supplementary Table S4). Phylogenetically, SdKPS clusters with other angiosperm CPS proteins that fall into the previously defined TPS-c clade, which contains only the ‘DXDD’ motif (Bohlmann *et al.*, 1998; Chen *et al.*, 2011). SdKPS is most closely related to two class II Lamiaceae diTPSs involved in specialized diterpenoid metabolism (Li *et al.*, 2012; Cui *et al.*, 2015) (Fig. 4). Four incomplete sequences, SdCPSL1–4 (Supplementary Tables S4, S5; Supplementary Fig. S1), grouped together with class II diTPSs that produce diterpenoids of ‘normal’ stereochemical configuration (Gao *et al.*, 2009; Caniard *et al.*, 2012; Sallaud *et al.*, 2012; Schalk *et al.*, 2012; Pateraki *et al.*, 2014; Zerbe *et al.*, 2014; Cui *et al.*, 2015) (Fig. 4). The other three proteins, SdKSL1–SdKSL3, which were each full length in the database, belong to a distinct group of the class I kaurene synthase-like (KSL) proteins of the TPS-e/f subfamily and possess the ‘DDXXD’ motif (Bohlmann *et al.*, 1998; Chen *et al.*, 2011) (Fig. 4). Because of the structure and absolute configuration of (–)-kolavenol and the fact that salvinorin diterpenoids are the most abundant diterpenoids in trichomes (Supplementary Fig. S2), the major class II diTPS contig, SdKPS, was deemed to be more likely to be involved in the biosynthesis of (–)-kolavenyl diphosphate and was chosen to be studied in detail.

### *In vitro* enzymatic activity of SdKPS

The open reading frame of *SdKPS* was directly amplified from *S. divinorum* glandular trichome cDNA. Full-length (Fl) *SdKPS* transcript appeared to encode a protein with a putative N-terminal transit peptide (Supplementary Fig. S3, 46 amino acids from the start codon). The truncated (Tr) recombinant SdKPS (putative transit peptide removed) was produced in *E. coli* and purified to near homogeneity using the C-terminally fused hexahistidine tag.

**Fig. 5.**
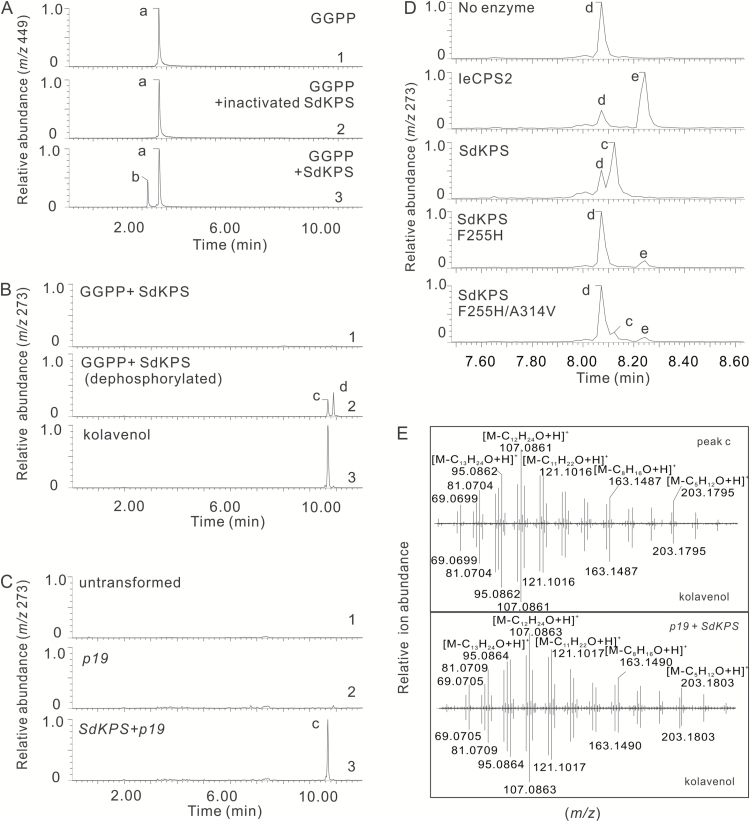
Functional characterization of SdKPS and relevant SdKPS mutants. (A) Extracted ion chromatogram (EIC) from LC-MS analysis under negative ionization mode of the non-dephosphorylated reaction product (peak b) of recombinant Tr-SdKPS using GGPP (peak a) as substrate (trace 3), compared with the reaction where there is no Tr-SdKPS (trace 1) or deactivated Tr-SdKPS (trace 2). The reactions are indicated at the upper-right corner of each panel. (B) EIC from LC-MS analysis under positive ionization mode of the dephosphorylated reaction product of recombinant Tr-SdKPS using GGPP as substrate (trace 2), compared with non-dephosphorylated reaction product (trace 1) and authentic standard of (–)-kolavenol (trace 3). Peak c, (–)-kolavenol; peak d, geranylgeraniol (GGOH, dephosphorylated GGPP). (C) Transient expression of Fl-SdKPS in leaves of *Nicotiana benthamiana*. EIC of metabolite extracts from *N. benthamiana* leaves (trace 1) or leaves after *Agrobacterium*-mediated transient co-expression of Fl-SdKPS with p19 for RNA-silencing suppression (trace 3) or leaves expressing p19 alone (trace 2) analyzed under the same LC-MS conditions applied in (B). (D) Effects of F255H and F255H/A314V mutations on the SdKPS product profile. EIC (*m*/*z* 273) from LC-MS analyses of the dephosphorylated products of reactions indicated in each panel. A different UPLC column from that in (A–C) was used, as detailed in the Materials and Methods. Peak e, *ent*-copalol. (E) Top panel: MS/MS spectrum of peak c produced by Tr-SdKPS after dephosphorylation as in Fig. 5B (trace 2), compared to that of authentic (–)-kolavenol. Bottom panel: comparison of MS/MS spectra of peak c detected in extract of *Fl-SdKPS*-transformed *N. benthamiana* leaves in Fig. 5C (trace 3) and that of authentic (–)-kolavenol.

*In vitro* assays were carried out with GGPP as the substrate and the products were analyzed by LC-MS to determine the enzymatic activity of Tr-SdKPS. The formation of a reaction product (Fig. 5A, peak b in trace 3) was detected in full assays, but not in controls (Fig. 5A, trace 1 and 2). The mass to charge ratio (*m*/*z*) of the corresponding singly charged deprotonated molecular ion species [M-H]^-^ was 449.1864 (see Supplementary Fig. S4B), which is consistent with the formula C_20_H_35_O_7_P_2_^–^ with a <0.1 ppm error, indicating that the product of Tr-SdKPS has the same elemental composition as GGPP (Supplementary Fig. S4). This is expected of (–)-KPP, which is formed by rearrangement and folding of GGPP. The dephosphorylated product of Tr-SdKPS (peak c in Fig. 5B, trace 2) was produced using non-specific calf intestinal phosphatase and identified as (–)-kolavenol based on its retention time, *m*/*z* value, and mass fragmentation pattern, which match perfectly those of the authentic standard (Fig. 5E). Kinetic analysis of Tr-SdKPS under optimized assay conditions (Supplementary Fig. S5) revealed that the reaction followed the Michaelis–Menten model. The determined *K*_M_ of 1.9 ± 0.66 µM and *k*_cat_ of 0.88 ± 0.11 s^-1^, with *k*_cat_/*K*_M_ of 4.7 × 10^5^ s^–1^ M^–1^ (Supplementary Fig. S6) are comparable to those of other characterized plant diTPSs with their native substrates (Prisic and Peters, 2007; Keeling *et al.*, 2008). Taken together, the *in vitro* assay results suggested that SdKPS acts as the monofunctional (–)-kolavenyl diphosphate synthase (KPS) initiating the biosynthesis of (–)-kolavenol in *S. divinorum*.

We also sought to elucidate the enzyme responsible for dephosphorylation of (–)-KPP in *S. divinorum* by expressing recombinant SdKSL1–3 for biochemical assessment. Both SdKSL1 and SdKSL3 were successfully expressed with their N-terminal transit peptides removed and were subsequently purified (see Supplementary Fig. S7). However, the co-incubation of SdKPS with either SdKSL1 or SdKSL3 and with GGPP as substrate did not result in any dephosphorylated product being formed. SdKSL2 was not expressed in *E. coli* to any detectable level under the same expression conditions as were used for SdKSL1 and SdKSL3, and the enzyme activity was no higher than the background. Therefore, the enzyme catalyzing the conversion of (–)-KPP into (–)-kolavenol in *S. divinorum* remains unknown.

### Transient expression of *SdKPS in planta*

*S. divinorum* is currently not amenable to genetic transformation. Therefore, the function of our candidate KPS was tested by infiltrating the leaves of *N. benthamiana* with *A. tumefaciens* strains carrying *Fl-SdKPS*. Protein p19 was co-expressed with *Fl-SdKPS* to improve the expression of the transgene (Voinnet *et al.*, 2003). (–)-Kolavenol could only be detected in extracts from leaves where the expression of *Fl-SdKPS* was expected to occur (Fig. 5C, trace 3; Fig. 5E), while it was not present in either untreated controls or in leaves infiltrated only with the p19 enhancer strain (Fig. 5C, trace 1 and 2), suggesting that *Fl-SdKPS* was successfully expressed and the encoded protein was catalytically active in *N. benthamiana* leaves. These results not only confirm the function of Fl-SdKPS as KPS, but also indicate that (–)-kolavenyl diphosphate can be dephosphorylated to (–)-kolavenol by an endogenous enzyme from *N. benthamiana* leaves. It has been previously suggested that endogenous phosphatases can convert the product of class II diTPSs into corresponding diterpenols in tobacco (Sallaud *et al.*, 2012; Zerbe *et al.*, 2014, 2015). Thus, *in vivo* functional characterization of Fl-SdKPS confirmed the results obtained *in vitro* with Tr-SdKPS.

### Developmental pattern of diterpenoid accumulation and *SdKPS* expression

The diterpenoid synthase required for (–)-kolavenol production is the first enzyme of the salvinorin A pathway (Fig. 1B). Therefore, analysis of relationships between accumulated diterpenes and *SdKPS* gene expression might further confirm or refute whether SdKPS plays the proposed role *in planta*. For this experiment, whole leaves of three developmental stages from five plants of the same age were used. Leaf pairs with distinct size and age were only collected from the main stem, with 1st pairs being the youngest (from the top node), the 3rd pairs being the middle (from the 3rd node), and the 5th pairs (from the 5th node) being the oldest at time of collection.

**Fig. 6.**
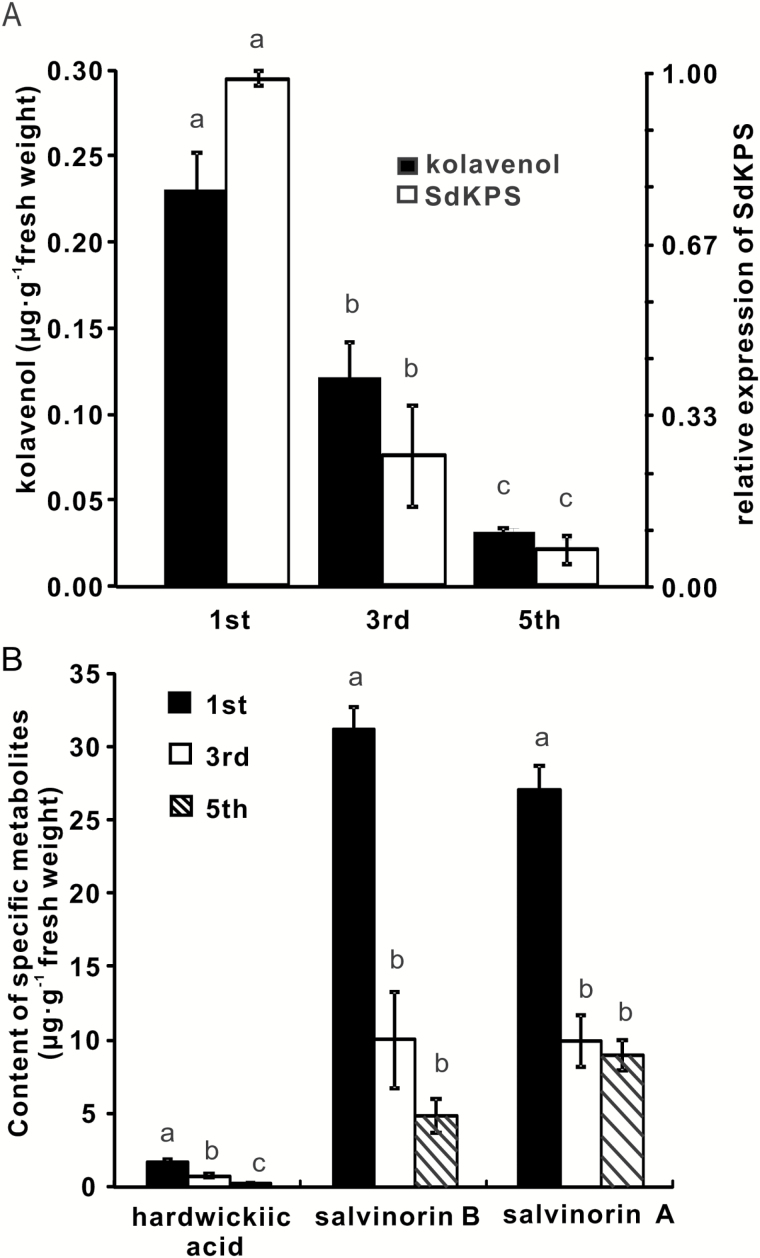
Accumulation of selected diterpenoids and transcript levels of *SdKPS* in *S. divinorum* leaves of different growth stages. (A) Comparison of (–)-kolavenol accumulation and *SdKPS* expression in the same leaf tissue of different growth stages. (B) Content of hardwickiic acid, salvinorin B, and salvinorin A in the same leaf tissue as in (A). Different letters indicate statistically significant differences in the metabolite concentrations in different leaf pairs as calculated using a one-way ANOVA test (*P*<0.05) with a Tukey HSD test. Results are the means of five biological replicates ±SE.

The salvinorin-related diterpenoids evaluated were (–)-kolavenol, hardwickiic acid, salvinorin B, and salvinorin A. (–)-Kolavenol and hardwickiic acid were monitored because they are closely connected to the KPS-catalyzed reaction and are early intermediates in the pathway, while salvinorin A and B are the principal products of the pathway (Kutrzeba *et al.*, 2009*a*) and thus represent the overall flow through the metabolic pathway. For all four diterpenoids analyzed, the levels of individual compounds were highest in the youngest leaf pair and decreased as the leaves matured (Fig. 6), following one common pattern of biosynthesis in Lamiaceae peltate glandular trichomes. (–)-Kolavenol, the earliest detectable intermediate of salvinorin A biosynthesis, accumulated at lower concentrations (0.03–0.23 µg g^–1^) than the other three diterpenoids, potentially reflecting its efficient conversion into compounds downstream in the pathway. The concentrations of hardwickiic acid were slightly higher than those of (–)-kolavenol (0.25–1.66 µg g^–1^) through the monitored developmental time course. Salvinorins A and B accumulated at much higher levels. The concentrations of both compounds decreased abruptly in the 3rd leaf pairs compared to the 1st, and then leveled off (Fig. 6B). In parallel with metabolite levels, the expression of SdKPS was highest in young leaves and decreased dramatically as the leaves aged. The difference of expression was 4-fold between the 1st and 3rd pair as well as between the 3rd and 5th pair (Fig. 6A). Although the transcript abundance may not reflect the enzymatic activity in the glands, the relationship of the differences between metabolite profiles and expression of *SdKPS* is in line with its proposed physiological role in *S. divinorum*.

### The basis for the product specificity of SdKPS

Although SdKPS is phylogenetically closely related to several characterized plant CPSs (Fig. 4), it exhibits a distinct function. Of the currently known CPSs, SdKPS shares the highest protein identity of 72% with IeCPS2 from *Isodon eriocalyx* (Li *et al.*, 2012). The differences between these proteins are sufficient to direct IeCPS2 to deprotonate the labda-13*E*-en-8-yl^+^ diphosphate at C-17, whereas SdKPS first proceeds through a series of 1,2-hydride and methyl shifts, and concludes with a deprotonation at C-3, resulting in (–)-KPP (Fig. 1A). A comparison of these two proteins appeared, therefore, to be a productive approach to identify the residues of SdKPS that mediate its product specificity. The predicted protein structure of SdKPS was first aligned with that of IeCPS2, and divergent residues within 4- and 8-Å spheres around C-8 of the substrate analogue in the structure of CPS from *Arabidopsis thaliana* were identified (Protein Data Bank: 3PYA) (Köksal *et al.*, 2011, 2014) and superimposed on our models by I-TASSER (Zhang, 2008). The sequence of SdKPS was then aligned with those of other related, functionally characterized plant CPSs (see Supplementary Fig. S3) to find residues unique to the active site of SdKPS (Table 3) and thus most likely to be involved in determining its product. In order to assess the effect of those residues on enzymatic function of SdKPS, they were replaced by the corresponding residues found in IeCPS2 by site-directed mutagenesis. The activities of the purified mutant proteins were then tested *in vitro*.

**Table 3.** *Divergent residues in SdKPS versus IeCPS2 models within 8 Å of the central carbon atom of the substrate analog in the structure of* ent*-CPS from* A. thaliana *and the corresponding residues in other plant* ent*-CPSs*

Radius, Å	Residue in						
	SdKPS	IeCPS2	PsCPS	CmCPS2	AtCPS	LsCPS	SrCPS
4	V200	I200	I	I	I	I	I
4	F255	H265	H	H	H	H	H
4	A314	V315	V	V	V	V	V
8	S369	V370	I	I	I	I	I
8	S372	T373	T	T	T	T	T
8	S373	A374	A	A	A	A	A
10	S402	C403	C	C	C	C	C

Three divergent residues, V200, F255, and A314 were identified within the predicted 4-Å sphere in SdKPS, corresponding to I200, H265, and V315 in IeCPS2. Most notably, the SdKPS:F255H mutant showed a complete switch of product specificity from (–)-KPP to *ent*-CPP (Fig. 5D). The other single mutations, V200I or A314V, on the other hand, did not change the product outcome (see Supplementary Fig. S8). Intriguingly, the double-mutant, A314V/F255H, led to the recovery of the ability to produce (–)-KPP while retaining the acquired function of forming *ent*-CPP (Fig. 5D). None of the divergent residues within the 4–10-Å sphere as shown in Table 3 redirected the catalytic routes from route a to route b (Fig. 1A), as revealed by product profiles of mutants SdKPS S369V, SdKPS S369V/S372T/S373A, and SdKPS S402C (Supplementary Fig. S8). Thus, it appears that only two residues modulate the product outcome of SdKPS relative to CPS.

## Discussion

### Biosynthesis of (–)-kolavenol in trichomes of *S. divinorum*

SdKPS was identified as a mono-product class II diTPS catalyzing the formation of (–)-KPP from GGPP as the first reaction in salvinorin A biosynthesis (Figs 1 and 5). Formation of (–)-KPP presumably starts from the protonation-initiated cyclization of GGPP, proceeds through a series of 1,2-hydride/methyl migrations on the initial intermediate labda-13-en-8-yl^+^ diphosphate, and is terminated by deprotonation (Fig. 1A, route a). The subsequent dephosphorylation of (–)-KPP could be catalyzed by a phosphatase or by a class I diTPS, a class of enzymes that mediates the formation of other labdane-related diterpenoids with or without subsequent hydroxylation of the dephosphorylated product (Zi *et al.*, 2014), including those with the clerodane backbone (Andersen-Ranberg *et al.*, 2016; Jia *et al.*, 2016).

As salvinorin A biosynthesis and storage primarily occurs in peltate trichomes in *S. divinorum* (Table 2; Figs 2 and 3), the concentration of diterpenoids in whole-leaf tissue is mainly determined by biosynthesis rates in individual trichomes and by trichome density. It is possible that the *de novo* biosynthesis of diterpenoids slows down or nearly ceases as the trichomes mature, as observed for monoterpenoid production in peppermint (McConkey *et al.*, 2000; Turner *et al.*, 2000).

### Identification of a highly specific enzyme catalyzing the formation of (–)-KPP

Several studies have demonstrated extreme product plasticity of class II (Criswell *et al.*, 2012; Potter *et al.*, 2014, 2015, 2016; Mafu *et al.*, 2015; Jia *et al.*, 2016) and class I diTPSs (Wilderman and Peters, 2007; Xu *et al.*, 2007*b*; Keeling *et al.*, 2008; Morrone *et al.*, 2008; Zerbe *et al.*, 2012; Irmisch *et al.*, 2015), providing evidence for the crucial role of neo-functionalization in the rise of diterpenoid chemical diversity. A very recent structure–function investigation of the catalytic histidine in AtCPS found that its substitution for the aromatic residues tyrosine or phenylalanine causes the enzyme to produce two novel products, one of them (–)-KPP (Potter *et al.*, 2015). Moreover, the reciprocal mutation of phenylalanine to histidine in SdKPS (F255H), as demonstrated herein, completely converts its enzymatic activity to CPS.

**Fig. 7.**
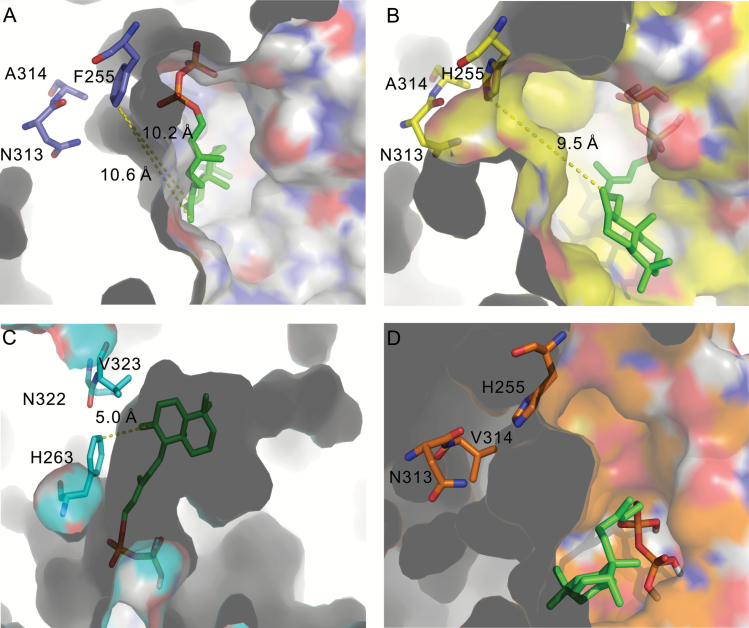
Models of active site cavities of SdKPS and related proteins. Active-site cavities are displayed for SdKPS (A), SdKPS:F255H (B), AtCPS:H263F (C), and SdKPS:F255H/A314V (D) modeled with labda-13*E*-en-8-yl^+^ diphosphate as the ligand. In (A–C) cavities are viewed from the entrance; in (D) the cavity is viewed from outside of the cavity wall. Carbon atoms in the backbone and surface of cavities are colored purple in (A), yellow in (B), cyan in (C), and orange in (D) to represent active sites from different enzymes. The grey shading represents the protein structure outside of the cavity side wall.

The substitution with a histidine of F255 not only forms a catalytic base group present in plant CPSs (Potter *et al.*, 2014) for deprotonation at C-17 of labda-13*E*-en-8-yl^+^ diphosphate intermediate, the orientation and location of the intermediate in the active site was also altered, as revealed by molecular docking (Fig. 7). The distance from C-17 of the ligand to F255 in SdKPS is longer than that from C-17 to H255 in SdKPS:F255H (Fig. 7A, B). In addition, the substitution of alanine with valine at residue 314 partially restores the KPS activity of SdKPS:F255H, giving rise to production of both (–)-KPP and *ent*-CPP. Residue 314 is located in close proximity to F255 and N313 in the predicted active-site cavity of SdKPS, and it lines the surface of the cavity (Fig. 7D). The larger side chain of valine compared to alanine presumably leads to an alteration of the steric shape of the side of the reaction pocket and the positioning of the labda-13*E*-en-8-yl^+^ diphosphate intermediate. Thus, C-17 of the intermediate is not efficiently deprotonated by H255 in the active-site pocket of SdKPS:F255H/A314V, which enables hydride and methyl migrations in the backbone rearrangement that leads to (–)-KPP formation.

The functional conversions of SdKPS by F255H mutation and AtCPS by H263F (Potter *et al.*, 2016) also pinpoint the catalytic histidine as a critical structural element of class II diTPSs underlying diversification of labdane-related diterpenoid biosynthesis in plants. The histidine in plant CPSs belongs to the catalytic His-Asn dyad of CPSs (H255 and N313 in SdKPS:F255H) (Potter *et al.*, 2014). This His residue was conserved in all known plant CPSs producing *ent*-CPP, including the bifunctional diterpene cyclase (PpCPSKS) from the moss *Physcomitrella patens*, suggesting that it originated more than 400 million years ago (Hayashi *et al.*, 2006; Potter *et al.*, 2014; Zi *et al.*, 2014). A divergent residue at this position appears to underlie the neo-functionalization of class II diTPSs, which resulted in expansion of labdane diterpenoid diversity in the plant kingdom. This notion is supported by the presence of aromatic residues (phenylalanine or tyrosine) at positions corresponding to SdKPS F255 in many other class II diTPSs involved in specialized biosynthesis of various labdane-type diterpenoids from different angiosperm lineages (see Supplementary Table S6). Specifically, a tyrosine is found at the corresponding position in the only other identified plant (–)-KPP synthase, TwTPS14 (Andersen-Ranberg *et al.*, 2016), supporting the idea that a stable aromatic residue at this position is essential for production of (–)-KPP.

Besides F255, there must be other factors contributing to product specificity of SdKPS, as many other enzymes with the F/Y residue do not produce (–)-KPP (see Supplementary Table S6) and AtCPS:H263F produces a secondary product, *ent*-labda-13*E*-en-8α-ol diphosphate. The inability of SdKPS to produce *ent*-labda-13*E*-en-8α-ol diphosphate indicates a unique strategy for preventing access to water by C-8 of the intermediate, which AtCPS:H263F lacks (Potter *et al.*, 2015). Intriguingly, the size and shape of the predicted active-site cavity of SdKPS differ from those of AtCPS:H263F (Fig. 7A, C). The labda-13*E*-en-8-yl^+^ diphosphate intermediate could be stabilized in a different orientation, where C-8 is more distant from the residue at position 255 in the cavity of SdKPS (Fig. 7A, C). Although the crystal structure of SdKPS has not been solved experimentally, the docking results based on the predicted SdKPS cavity structure suggested that, along with the steric block by phenylalanine, the orientation and position of C-8 of the intermediate, probably determined by the shape and size of the active-site cavity, enable the full elimination of hydroxylation on C-8. A high-resolution crystal structure of SdKPS will help answer these questions.

### The evolution of kolavenol biosynthesis

SdKPS represents one of the very few examples of a diTPSs forming the clerodane backbone in plants. The only other known plant diTPS catalyzing a similar reaction is the very recently reported class II diTPS TwTPS14 from *Tripterygium wilfordii* (Celastraceae) (Andersen-Ranberg *et al.*, 2016). The low protein sequence identity (53%) and the phylogenetic relationship between SdKPS and TwTPS14 suggest that the ability to produce (–)-kolavenyl diphosphate arose independently in these two plant lineages. Phylogenetic analysis (Fig. 4) shows that SdKPS clusters together with other Lamiaceae diTPSs involved in specialized diterpenoid biosynthesis. On the other hand, TwTPS14 is much more closely related to functionally different diTPSs from *T. wilfordii* (Andersen-Ranberg *et al.*, 2016). The convergent or repeated evolution scenario (Pichersky and Gang, 2000; Pichersky and Lewinsohn, 2011) of kolavenol biosynthesis is also evident in the fact that a diTPS catalyzing the formation of (+)-KPP has recently been isolated from *Herpetosiphon aurantiacus*, a filamentous green non-sulfur bacterium producing the clerodane diterpenoid methylkolavelool (Nakano *et al.*, 2015). While it also contains the DXDD motif and is classified as a class II diTPS, the overall protein identity of the bacterial diTPS with SdKPS is less than 25%.

The independent evolution of kolavenol biosynthesis in the plant kingdom might be driven by the selective pressure provided by the insect anti-feedant activity of clerodanes. Indeed, the higher concentration of the clerodane diterpenoids in younger leaves and insect anti-feedant activities of various clerodanes (Sosa *et al.*, 1994; Klein Gebbinck *et al.*, 2002; Rosselli *et al.*, 2004; Sivasubramanian *et al.*, 2013) suggest that they may serve to protect young susceptible tissues from herbivore attack.

## Supplementary Material

Supplementary Data

## References

[CIT0001] Andersen-Ranberg J Kongstad KT Nielsen MT 2016 Expanding the landscape of diterpene structural diversity through stereochemically controlled combinatorial biosynthesis. Angewandte Chemie 55, 1–6. 10.1002/anie.201510650 PMC4755150 26749264

[CIT0002] Berim A Hyatt DC Gang DR 2012 A set of regioselective O-methyltransferases gives rise to the complex pattern of methoxylated flavones in sweet basil. Plant Physiology 160, 1052–1069. 22923679 10.1104/pp.112.204164 PMC3461529

[CIT0003] Bigham AK Munro TA Rizzacasa MA Robins-Browne RM 2003 Divinatorins A-C, new neoclerodane diterpenoids from the controlled sage *Salvia divinorum*. Journal of Natural Products 66, 1242–1244. 14510607 10.1021/np030313i

[CIT0004] Bohlmann J Meyer-Gauen G Croteau R 1998 Plant terpenoid synthases: molecular biology and phylogenetic analysis. Proceedings of the National Academy of Sciences, USA 95, 4126–4133. 10.1073/pnas.95.8.4126 PMC22453 9539701

[CIT0005] Brückner K Božić D Manzano D 2014 Characterization of two genes for the biosynthesis of abietane-type diterpenes in rosemary (*Rosmarinus officinalis*) glandular trichomes. Phytochemistry 101, 52–64. 24569175 10.1016/j.phytochem.2014.01.021

[CIT0006] Butelman ER Kreek MJ 2015 Salvinorin A, a kappa-opioid receptor agonist hallucinogen: pharmacology and potential template for novel pharmacotherapeutic agents in neuropsychiatric disorders. Frontiers in Pharmacology 6, 190. 26441647 10.3389/fphar.2015.00190 PMC4561799

[CIT0007] Caniard A Zerbe P Legrand S Cohade A Valot N Magnard JL Bohlmann J Legendre L 2012 Discovery and functional characterization of two diterpene synthases for sclareol biosynthesis in *Salvia sclarea* (L.) and their relevance for perfume manufacture. BMC Plant Biology 12, 119. 22834731 10.1186/1471-2229-12-119 PMC3520730

[CIT0008] Chen F Tholl D Bohlmann J Pichersky E 2011 The family of terpene synthases in plants: a mid-size family of genes for specialized metabolism that is highly diversified throughout the kingdom. The Plant Journal 66, 212–229. 21443633 10.1111/j.1365-313X.2011.04520.x

[CIT0009] Criswell J Potter K Shephard F Beale MH Peters RJ 2012 A single residue change leads to a hydroxylated product from the class II diterpene cyclization catalyzed by abietadiene synthase. Organic Letters 14, 5828–5831. 23167845 10.1021/ol3026022 PMC3518578

[CIT0010] Cui G Duan L Jin B 2015 Functional divergence of diterpene syntheses in the medicinal plant *Salvia miltiorrhiza*. Plant Physiology 169, 1607–1618. 26077765 10.1104/pp.15.00695 PMC4634056

[CIT0011] Earley KW Haag JR Pontes O Opper K Juehne T Song K Pikaard CS 2006 Gateway-compatible vectors for plant functional genomics and proteomics. The Plant Journal 45, 616–629. 16441352 10.1111/j.1365-313X.2005.02617.x

[CIT0012] Felsenstein J 2010 Confidence limits on phylogenies: an approach using the bootstrap. Evolution 39, 783–791. 10.1111/j.1558-5646.1985.tb00420.x 28561359

[CIT0013] Gang DR Wang J Dudareva N Nam KH Simon JE Lewinsohn E Pichersky E 2001 An investigation of the storage and biosynthesis of phenylpropenes in sweet basil. Plant Physiology 125, 539–555. 11161012 10.1104/pp.125.2.539 PMC64856

[CIT0014] Gao W Hillwig ML Huang L Cui G Wang X Kong J Yang B Peters RJ 2009 A functional genomics approach to tanshinone biosynthesis provides stereochemical insights. Organic Letters 11, 5170–5173. 19905026 10.1021/ol902051v PMC2776380

[CIT0015] Hayashi K Kawaide H Notomi M Sakigi Y Matsuo A Nozaki H 2006 Identification and functional analysis of bifunctional *ent*-kaurene synthase from the moss *Physcomitrella patens*. FEBS Letters 580, 6175–6181. 17064690 10.1016/j.febslet.2006.10.018

[CIT0016] He R Kim MJ Nelson W 2012 Next-generation sequencing-based transcriptomic and proteomic analysis of the common reed, *Phragmites australis* (Poaceae), reveals genes involved in invasiveness and rhizome specificity. American Journal of Botany 99, 232–247. 22301892 10.3732/ajb.1100429

[CIT0017] Irmisch S Müller AT Schmidt L Günther J Gershenzon J Köllner TG 2015 One amino acid makes the difference: the formation of *ent*-kaurene and 16α-hydroxy-*ent*-kaurane by diterpene synthases in poplar. BMC Plant Biology 15, 262. 26511849 10.1186/s12870-015-0647-6 PMC4625925

[CIT0018] Jia M Potter KC Peters RJ 2016 Extreme promiscuity of a bacterial and a plant diterpene synthase enables combinatorial biosynthesis. Metabolic Engineering 37, 24–34. 27060773 10.1016/j.ymben.2016.04.001 PMC4907819

[CIT0019] Keeling CI Dullat HK Yuen M Ralph SG Jancsik S Bohlmann J 2010 Identification and functional characterization of monofunctional *ent*-copalyl diphosphate and *ent*-kaurene synthases in white spruce reveal different patterns for diterpene synthase evolution for primary and secondary metabolism in gymnosperms. Plant Physiology 152, 1197–1208. 20044448 10.1104/pp.109.151456 PMC2832265

[CIT0020] Keeling CI Weisshaar S Lin RPC Bohlmann J 2008 Functional plasticity of paralogous diterpene synthases involved in conifer defense. Proceedings of the National Academy of Sciences, USA 105, 1085–1090. 10.1073/pnas.0709466105 PMC2242725 18198275

[CIT0021] Klein Gebbinck EA Jansen BJ de Groot A 2002 Insect antifeedant activity of clerodane diterpenes and related model compounds. Phytochemistry 61, 737–770. 12453568 10.1016/s0031-9422(02)00174-7

[CIT0022] Köksal M Hu H Coates RM Peters RJ Christianson DW 2011 Structure and mechanism of the diterpene cyclase *ent*-copalyl diphosphate synthase. Nature Chemical Biology 7, 431–433. 21602811 10.1038/nchembio.578 PMC3118866

[CIT0023] Köksal M Potter K Peters RJ Christianson DW 2014 1.55Å-resolution structure of *ent*-copalyl diphosphate synthase and exploration of general acid function by site-directed mutagenesis. Biochimica et Biophysica Acta 1840, 184–190. 24036329 10.1016/j.bbagen.2013.09.004 PMC3859867

[CIT0024] Kolak U Kabouche A Oztürk M Kabouche Z Topçu G Ulubelen A 2009 Antioxidant diterpenoids from the roots of *Salvia barrelieri*. Phytochemical Analysis 20, 320–327. 19402189 10.1002/pca.1130

[CIT0025] Kutrzeba L Dayan FE Howell J Feng J Giner JL Zjawiony JK 2007 Biosynthesis of salvinorin A proceeds via the deoxyxylulose phosphate pathway. Phytochemistry 68, 1872–1881. 17574635 10.1016/j.phytochem.2007.04.034 PMC2065853

[CIT0026] Kutrzeba LM Ferreira D Zjawiony JK 2009a Salvinorins J from *Salvia divinorum*: mutarotation in the neoclerodane system. Journal of Natural Products 72, 1361–1363. 19473009 10.1021/np900181q

[CIT0027] Kutrzeba LM Zjawiony JK Koo HJ McDowell E Laurenzi A Gang DR Dayan FE 2009b Biosynthesis of salvinorin A: overexpression and biochemical characterization of carboxy methyltransferase from EST of *Salvia divinorum* glands. Planta Medica 75, 431.

[CIT0028] Lange BM Turner GW 2013 Terpenoid biosynthesis in trichomes—current status and future opportunities. Plant Biotechnology Journal 11, 2–22. 22979959 10.1111/j.1467-7652.2012.00737.x

[CIT0029] Larkin MA Blackshields G Brown NP 2007 Clustal W and Clustal X version 2.0. Bioinformatics 23, 2947–2948. 17846036 10.1093/bioinformatics/btm404

[CIT0030] Li JL Chen QQ Jin QP Gao J Zhao PJ Lu S Zeng Y 2012 IeCPS2 is potentially involved in the biosynthesis of pharmacologically active Isodon diterpenoids rather than gibberellin. Phytochemistry 76, 32–39. 22284743 10.1016/j.phytochem.2011.12.021

[CIT0031] Mafu S Potter KC Hillwig ML Schulte S Criswell J Peters RJ 2015 Efficient heterocyclisation by (di)terpene synthases. Chemical Communications 51, 13485–13487. 26214384 10.1039/c5cc05754j PMC4543578

[CIT0032] Marchant NJ Whitaker LR Bossert JM 2016 Behavioral and physiological effects of a novel kappa-opioid receptor-based DREADD in rats. Neuropsychopharmacology 41, 402–409. 26019014 10.1038/npp.2015.149 PMC5130116

[CIT0033] McConkey ME Gershenzon J Croteau RB 2000 Developmental regulation of monoterpene biosynthesis in the glandular trichomes of peppermint. Plant Physiology 122, 215–224. 10631265 10.1104/pp.122.1.215 PMC58860

[CIT0034] Michavila A Delatorre MC Rodriguez B 1986 20-nor-abietane and rearranged abietane diterpenoids from the root of *Salvia argentea*. Phytochemistry 25, 1935–1937.

[CIT0035] Morrone D Xu M Fulton DB Determan MK Peters RJ 2008 Increasing complexity of a diterpene synthase reaction with a single residue switch. Journal of the American Chemical Society 130, 5400–5401. 18366162 10.1021/ja710524w

[CIT0036] Munro TA Rizzacasa MA 2003 Salvinorins D–F, new neoclerodane diterpenoids from *Salvia divinorum*, and an improved method for the isolation of salvinorin A. Journal of Natural Products 66, 703–705. 12762813 10.1021/np0205699

[CIT0037] Nakano C Oshima M Kurashima N Hoshino T 2015 Identification of a new diterpene biosynthetic gene cluster that produces *O*-methylkolavelool in *Herpetosiphon aurantiacus*. ChemBioChem 16, 772–781. 25694050 10.1002/cbic.201402652

[CIT0038] Nozawa M Suka Y Hoshi T Suzuki T Hagiwara H 2008 Total synthesis of the hallucinogenic neoclerodane diterpenoid salvinorin A. Organic Letters 10, 1365–1368. 18311991 10.1021/ol800101v

[CIT0039] Pateraki I Andersen-Ranberg J Hamberger B 2014 Manoyl oxide (13R), the biosynthetic precursor of forskolin, is synthesized in specialized root cork cells in *Coleus forskohlii*. Plant Physiology 164, 1222–1236. 24481136 10.1104/pp.113.228429 PMC3938615

[CIT0040] Peters RJ 2010 Two rings in them all: the labdane-related diterpenoids. Natural Product Reports 27, 1521–1530. 20890488 10.1039/c0np00019a PMC3766046

[CIT0041] Pichersky E Gang DR 2000 Genetics and biochemistry of secondary metabolites in plants: an evolutionary perspective. Trends in Plant Science 5, 439–445. 11044721 10.1016/s1360-1385(00)01741-6

[CIT0042] Pichersky E Lewinsohn E 2011 Convergent evolution in plant specialized metabolism. Annual Review of Plant Biology 62, 549–566. 10.1146/annurev-arplant-042110-103814 21275647

[CIT0043] Pittaluga A Olivero G Di Prisco S Merega E Bisio A Romussi G Grilli M Marchi M 2013 Effects of the neoclerodane Hardwickiic acid on the presynaptic opioid receptors which modulate noradrenaline and dopamine release in mouse central nervous system. Neurochemistry International 62, 354–359. 23357481 10.1016/j.neuint.2013.01.016

[CIT0044] Potter K Criswell J Zi J Stubbs A Peters RJ 2014 Novel product chemistry from mechanistic analysis of *ent*-copalyl diphosphate synthases from plant hormone biosynthesis. Angewandte Chemie 53, 7198–7202. 24862907 10.1002/anie.201402911 PMC4113509

[CIT0045] Potter KC Jia M Hong YJ Tantillo D Peters RJ 2016 Product rearrangement from altering a single residue in the rice *syn*-copalyl diphosphate synthase. Organic Letters 18, 1060–1063. 26878189 10.1021/acs.orglett.6b00181 PMC4782720

[CIT0046] Potter KC Zi J Hong YJ Schulte S Malchow B Tantillo DJ Peters RJ 2015 Blocking deprotonation with retention of aromaticity in a plant *ent*-copalyl diphosphate synthase leads to product rearrangement. Angewandte Chemie 55, 634–638. 26603275 10.1002/anie.201509060 PMC4768914

[CIT0047] Prisic S Peters RJ 2007 Synergistic substrate inhibition of *ent*-copalyl diphosphate synthase: a potential feed-forward inhibition mechanism limiting gibberellin metabolism. Plant Physiology 144, 445–454. 17384166 10.1104/pp.106.095208 PMC1913771

[CIT0048] Rosselli S Maggio A Piozzi F Simmonds MS Bruno M 2004 Extremely potent antifeedant neo-clerodane derivatives of scutecyprol A. Journal of Agricultural and Food Chemistry 52, 7867–7871. 15612769 10.1021/jf048532c

[CIT0049] Roth BL Baner K Westkaemper R Siebert D Rice KC Steinberg S Ernsberger P Rothman RB 2002 Salvinorin A: a potent naturally occurring nonnitrogenous κ opioid selective agonist. Proceedings of the National Academy of Sciences, USA 99, 11934–11939. 10.1073/pnas.182234399 PMC129372 12192085

[CIT0050] Roy A Kucukural A Zhang Y 2010 I-TASSER: a unified platform for automated protein structure and function prediction. Nature Protocols 5, 725–738. 20360767 10.1038/nprot.2010.5 PMC2849174

[CIT0051] Sadowski J Gasteiger J Klebe G 1994 Comparison of automatic three-dimensional model builders using 639 X-ray structures. Journal of Chemical Information and Modeling 34, 1000–1008.

[CIT0052] Saitou N Nei M 1987 The neighbor-joining method: a new method for reconstructing phylogenetic trees. Molecular Biology and Evolution 4, 406–425. 3447015 10.1093/oxfordjournals.molbev.a040454

[CIT0053] Sallaud C Giacalone C Töpfer R Goepfert S Bakaher N Rösti S Tissier A 2012 Characterization of two genes for the biosynthesis of the labdane diterpene Z-abienol in tobacco (*Nicotiana tabacum*) glandular trichomes. Plant Journal 72, 1–17. 22672125 10.1111/j.1365-313X.2012.05068.x

[CIT0054] Schalk M Pastore L Mirata MA Khim S Schouwey M Deguerry F Pineda V Rocci L Daviet L 2012 Toward a biosynthetic route to sclareol and amber odorants. Journal of the American Chemical Society 134, 18900–18903. 23113661 10.1021/ja307404u

[CIT0055] Schmittgen TD Livak KJ 2008 Analyzing real-time PCR data by the comparative *C*_T_ method. Nature Protocols 3, 1101–1108. 18546601 10.1038/nprot.2008.73

[CIT0056] Siebert DJ 2004 Localization of salvinorin A and related compounds in glandular trichomes of the psychoactive sage, *Salvia divinorum*. Annals of Botany 93, 763–771. 15087301 10.1093/aob/mch089 PMC4242294

[CIT0057] Simoes F Michavila A Rodriguez B Garciaalvarez MC Hasan M 1986 A quinone methide diterpenoid from the root of *Salvia moorcraftiana*. Phytochemistry 25, 755–756.

[CIT0058] Simonson B Morani AS Ewald AW Walker L Kumar N Simpson D Miller JH Prisinzano TE Kivell BM 2015 Pharmacology and anti-addiction effects of the novel κ opioid receptor agonist Mesyl Sal B, a potent and long-acting analogue of salvinorin A. British Journal of Pharmacology 172, 515–531. 24641310 10.1111/bph.12692 PMC4292965

[CIT0059] Sivasubramanian A Gadepalli Narasimha KK Rathnasamy R Campos AM 2013 A new antifeedant clerodane diterpenoid from *Tinospora cordifolia*. Natural Product Research 27, 1431–1436. 22946632 10.1080/14786419.2012.722088

[CIT0060] Sosa ME Tonn CE Giordano OS 1994 Insect antifeedant activity of clerodane diterpenoids. Journal of Natural Products 57, 1262–1265. 7798961 10.1021/np50111a012

[CIT0061] Sparkes IA Runions J Kearns A Hawes C 2006 Rapid, transient expression of fluorescent fusion proteins in tobacco plants and generation of stably transformed plants. Nature Protocols 1, 2019–2025. 17487191 10.1038/nprot.2006.286

[CIT0062] Steffen C Thomas K Huniar U Hellweg A Rubner O Schroer A 2010 TmoleX–a graphical user interface for TURBOMOLE. Journal of Computational Chemistry 31, 2967–2970. 20928852 10.1002/jcc.21576

[CIT0063] Studier FW 2005 Protein production by auto-induction in high density shaking cultures. Protein Expression and Purification 41, 207–234. 15915565 10.1016/j.pep.2005.01.016

[CIT0064] Tamura K Stecher G Peterson D Filipski A Kumar S 2013 MEGA6: molecular evolutionary genetics analysis version 6.0. Molecular Biology and Evolution 30, 2725–2729. 24132122 10.1093/molbev/mst197 PMC3840312

[CIT0065] Tejeda HA Shippenberg TS Henriksson R 2012 The dynorphin/κ-opioid receptor system and its role in psychiatric disorders. Cellular and Molecular Life Sciences 69, 857–896. 22002579 10.1007/s00018-011-0844-x PMC11114766

[CIT0066] Tokoroyama T 2000 Synthesis of clerodane diterpenoids and related compounds - Stereoselective construction of the decalin skeleton with multiple contiguous stereogenic centers. Synthesis, 2000, 611–633.

[CIT0067] Turner GW Gershenzon J Croteau RB 2000 Distribution of peltate glandular trichomes on developing leaves of peppermint. Plant Physiology 124, 655–664. 11027715 10.1104/pp.124.2.655 PMC59171

[CIT0068] Ulubelen A Evren N Tuzlaci E Johansson C 1988 Diterpenoids from the roots of *Salvia hypargeia*. Journal of Natural Products 51, 1178–1183. 3236010 10.1021/np50060a021

[CIT0069] Valdes LJ III Butler WM Hatfield GM Paul AG Koreeda M 1984 Divinorin A, a psychotropic terpenoid, and divinorin B from the hallucinogenic Mexican mint, *Salvia divinorum*. Journal of Organic Chemistry 49, 4716–4720.

[CIT0070] Valdés LJ III Chang HM Visger DC Koreeda M 2001 Salvinorin C, a new neoclerodane diterpene from a bioactive fraction of the hallucinogenic Mexican mint *Salvia divinorum*. Organic Letters 3, 3935–3937. 11720573 10.1021/ol016820d

[CIT0071] Voinnet O Rivas S Mestre P Baulcombe D 2003 An enhanced transient expression system in plants based on suppression of gene silencing by the p19 protein of tomato bushy stunt virus. The Plant Journal 33, 949–956. 12609035 10.1046/j.1365-313x.2003.01676.x

[CIT0072] Wasson RG 1962 A new Mexican psychotropic drug from the mint family. Botanical Museum Leaflets, Harvard University 20, 77–84.

[CIT0073] Wilderman PR Peters RJ 2007 A single residue switch converts abietadiene synthase into a pimaradiene specific cyclase. Journal of the American Chemical Society 129, 15736–15737. 18052062 10.1021/ja074977g PMC2525807

[CIT0074] Xu M Wilderman PR Morrone D Xu J Roy A Margis-Pinheiro M Upadhyaya NM Coates RM Peters RJ 2007a Functional characterization of the rice kaurene synthase-like gene family. Phytochemistry 68, 312–326. 17141283 10.1016/j.phytochem.2006.10.016

[CIT0075] Xu M Wilderman PR Peters RJ 2007b Following evolution’s lead to a single residue switch for diterpene synthase product outcome. Proceedings of the National Academy of Sciences, USA 104, 7397–7401. 10.1073/pnas.0611454104 PMC1855280 17456599

[CIT0076] Zerbe P Chiang A Bohlmann J 2012 Mutational analysis of white spruce (*Picea glauca*) *ent*-kaurene synthase (PgKS) reveals common and distinct mechanisms of conifer diterpene synthases of general and specialized metabolism. Phytochemistry 74, 30–39. 22177479 10.1016/j.phytochem.2011.11.004

[CIT0077] Zerbe P Chiang A Dullat H O’Neil-Johnson M Starks C Hamberger B Bohlmann J 2014 Diterpene synthases of the biosynthetic system of medicinally active diterpenoids in *Marrubium vulgare*. Plant Journal 79, 914–927. 24990389 10.1111/tpj.12589

[CIT0078] Zerbe P Hamberger B Yuen MM Chiang A Sandhu HK Madilao LL Nguyen A Hamberger B Bach SS Bohlmann J 2013 Gene discovery of modular diterpene metabolism in nonmodel systems. Plant Physiology 162, 1073–1091. 23613273 10.1104/pp.113.218347 PMC3668041

[CIT0079] Zerbe P Rodriguez SM Mafu S Chiang A Sandhu HK O’Neil-Johnson M Starks CM Bohlmann J 2015 Exploring diterpene metabolism in non-model species: transcriptome-enabled discovery and functional characterization of labda-7,13*E*-dienyl diphosphate synthase from *Grindelia robusta*. Plant Journal 83, 783–793. 26119826 10.1111/tpj.12925

[CIT0080] Zhang Y 2008 I-TASSER server for protein 3D structure prediction. BMC Bioinformatics 9, 40. 18215316 10.1186/1471-2105-9-40 PMC2245901

[CIT0081] Zi J Mafu S Peters RJ 2014 To gibberellins and beyond! Surveying the evolution of (di)terpenoid metabolism. Annual Review of Plant Biology 65, 259–286. 10.1146/annurev-arplant-050213-035705 PMC4118669 24471837

