# Supplementary material for: A (–)-kolavenyl diphosphate synthase catalyzes the first step of salvinorin A biosynthesis in Salvia divinorum
Source: J Exp Bot. 2017 Feb 15;68(5):1109–22. doi: 10.1093/jxb/erw493 (PMC5441855; doi:10.1093/jxb/erw493)
Supplement: Supplementary Data [file erw493_Supplementary_Data.zip › supplementary_tables_S1_S6_figures_S1_S8.pdf]

Supplementary Tables:

Supplementary Table S1
All primers used in this study, as indicated in Materials and Methods.

SdKPS-FULL-F	ATGTCATTCGCCACCTCCCT
SdKPS-truncation-F	ATGCTCAAATCAAAGGCTGGGA
SdKPS-exp-R	GACAATTTTTTCAAACAATACTTTG
SdKPS-his-R	ATGGTGATGGTGATGATGGACAATTTTTTCAAACAATACTTTG
SdKPS-F	CATCAGGGAAGGCAAAGTACA
SdKPS-R	ACCAATATCTTCCACGAGCG
EF-1-F	CCAAGAGACCCACAGACAAG
EF-1-R	GACCATACCAGGCTTCAGTAG
S369V	GGCACGAAATATGTGTGTCCAAGATGTTGACGACTCCTCAA
S402C	AAGGTGGACAATTCTGCTGCATACCAGGACAGATG
F255H	AAAGCCAACATCACTACTCCACAGCTTGGAAGGCATGGAA
A314V	ACGGTGGAGTTCCAAATGTATACCCTGTCGACATG
V200I	CGAGGAGCTCATGCCCATAGGATTCGAAATCGC
S369V/S372T/S373A	TCCAAGATGTTGACGACACCGCAATGGGATTCAGGCTG
SdKSL1-F	CTCGAGCATGTTACAGTCCAAGGATTTC
SdKSL1-R	GGATCCTCATTTGCCACTCACATTGTTAG
SdKSL2-F	GAGCTCGCCATCGCCAAAAAGTCATATG
SdKSL2-R	GCGGCCGCTTAACTCGAAAGTGTAAGAGGCTCG
SdKSL3-F	CTCGAGACTACTTCACCAAAAATCACCTC
SdKSL3-R	GGATCCTCACAACTTTTGCTCCTTGAGAA

Supplementary Table S2

The expression of *EF-1* in leaf tissues of different growth stage presented as 2^{-C_T}. Results are means of five biological replicates ± standard error.

	1st leaf pair	3rd leaf pair	5th leaf pair
2^{-C_T}	5.9E-7±5.7E-8	4.8E-7±7.6 E-8	6.0E-7±1.4E-8

Supplementary Table S3
List of the names and accession numbers of proteins displayed in Fig. 4.

Abbreviation	Species and Enzyme Name	Accession #
AtCPS	*Arabidopsis thaliana ent*-copalyl diphosphate synthase	NP_192187.1
AtKS	*Arabidopsis thaliana ent*-kaurene synthase	AAC39443
CaCPS	*Coffea arabica ent*-copalyl diphosphate synthase	ACQ99373.1
CcCLS	*Cistus creticus* copal-8-ol synthase	ADJ93862
CfTPS1	*Coleus forskohlii* copalyl diphosphate synthase	KF444506
CfTPS4	*Coleus forskohlii* milliradiene synthase	KF444509
CfTPS2	*Coleus forskohlii* Copal-8-ol diphosphate synthase	KF444507
CfTPS3	*Coleus forskohlii* milliradiene synthase	KF444508
CmKS	*Cucurbita maxima Ent*-kaurene synthase	Q39548
CsCPS	*Croton sublyratus* copalyl diphosphate synthase	BAA95612.1
EPTPS7	*Euphorbia peplus ent*-copalyl diphosphate synthase	KC7022396
GrTPS1	*Grindelia robusta* Copal-8-ol diphosphate synthase	AGN70887
GrTPS2	*Grindelia robusta* labd-7,13E-dienyl diphosphate synthase	AKP96361
HvCPS	*Hordeum vulgare* copalyl diphosphate synthase-like protein	AAT49065
IeCPS2	*Isodon eriocalyx* copalyl diphosphate synthase	AEP03175
LsCPS	*Lactuca sativa* copalyl diphosphate synthase	BAB12440
LsKS	*Lactuca sativa ent*-kaurene synthase	BAB12441
MvCPS1	*Marrubium vulgare* peregrinol diphosphate synthase	KJ584450
MvCPS3	*Marrubium vulgare* copalyl diphosphate synthase	KJ584452
MvCPS5	*Marrubium vulgare* 9, 13-epoxy-labd-14-en synthase	KJ584454
NtABS	*Nicotiana tabacum cis*-abienol synthase	CCD33019.1
NtCPS2	*Nicotiana tabacum* 8-hydroxy-copalyl diphosphate synthase	G3CCC0
OsCPS1	*Oryza sativa ent*-copalyl diphosphate synthase	Q6ET36
OsCPS4	*Oryza sativa syn*-copalyl diphosphate synthase	Q0JF02
OsKSL6	*Oryza sativa* Japonica Group *iso*-kaurene synthase	ABH10733.1
OsKSL7	*Oryza sativa* Indica Group *ent*-cassa-12, 15-diene synthase	Q00G37
PpCPS/KS	*Physcomitrella patens ent*-kaurene synthase	BAF61135
PsCPS	*Pisum sativum ent*-copalyl diphosphate synthase	O04408
ScdCPS	*Scoparia dulcis ent*-copalyl diphosphate synthase	BAD91286
ShSBS	*Solanum habrochaites* santalene and bergamotene synthase	ACJ38409
SlCPS	*Solanum lycopersicum ent*-copalyl diphosphate synthase	BAA84918
SlPHS	*Solanum lycopersicum* terpene synthase	ACO56896.1
SmCPS1	*Salvia miltiorrhiza f. alba* copalyl diphosphate synthase	AHJ59321
SmCPS2	*Salvia miltiorrhiza f. alba* copalyl diphosphate synthase	AHJ59322
SmCPS4	*Salvia miltiorrhiza f. alba* Copal-8-ol diphosphate synthase	AKN91186
SmCPS5	*Salvia miltiorrhiza f. alba ent*-copalyl diphosphate synthase 5	AKN91186
SmKSL1	*Salvia miltiorrhiza* kaurene synthase	ABV08817

SmKSL2	*Salvia miltiorrhiza ent*-copalyl diphosphate synthase	KC814643
SrCPS	*Stevia rebaudiana* copalyl diphosphate synthase	AAB87091
SrKS1	*Stevia rebaudiana* kaurene synthase	AAD34294
SsLPS	*Salvia sclarea* Copal-8-ol diphosphate synthase	AET21247
SsSS	*Salvia sclarea* sclareol synthase	AET21246.1
TwTPS14	*Trypterygium wilfordii* clerodienyl diphosphate synthase	KP889111
TwTPS21	*Trypterygium wilfordii* Copal-8-ol diphosphate synthase	KP889112
TwTPS28	*Trypterygium wilfordii* class II diterpene synthase	KP889113
ZmCPS	*Zea mays ent*-copalyl diphosphate synthase	AAA73960
ZmKS	*Zea mays ent*-kaurene synthase B	NP_001148059

Supplementary Table S4
Expression of candidate SdKPS and related genes
Values are reads per kilobase of transcript per million mapped reads (RPKM).

**Contigs**	**Expression RPKM**
SdKPS	207
SdCPSL1	31.1
SdCPSL2	1.15
SdCPSL3	1.23
SdCPSL4	1.24
SdKSL1	7.55
SdKSL2	5.99
SdKSL3	2.82

Supplementary Table S5
The top three matches of SdCPSL2-4 in UniProtKB/Swiss-Prot identified by BLAST search. Amino acid identity between each pair of query and subject sequence is shown in parentheses.

***S. divinorum* contigs**	**Top hits in UniProtKB/Swiss-Prot**		
	**1st**	**2nd**	**3rd**
SdCPSL2	CcCLS (51%)	PsCPS (48%)	AtCPS (47%)
SdCPSL3	CcCLS (52%)	PsCPS (50%)	OsCPS4 (49%)
SdCPSL4	NtCPS2 (52%)	AtCPS (51%)	PsCPS (51%)

Supplementary Table S6

Characterized class II diTPSs that contain aromatic residues (phenylalanine or tyrosine) at the same position as SdKPS F255 for specialized biosynthesis of various labdane-type diterpenoids from different angiosperm lineages.

diTPS	Species	residue	product	Accession NO.	reference
**SdKPS**	*Salvia divinorum*	F (255)	(-)-kolavenyl diphosphate	KX268505	This work
**SmCPSKSL1**	*Selaginella moellendorffii*	Y (168)	labd-7,13E-dien-15-ol	G1DGI7	Mafu et al., 2011
**SmCPS4**	*Salvia miltiorrhiza*	F (244)	labd-13-en-8-ol diphosphate	AKN91186	Cui et al., 2015
**GrTPS1**	*Grindelia robusta*	Y (240)	labd-13-en-8-ol diphosphate	AGN70887	Zerbe et al., 2013
**GrTPS2**	*G. robusta*	Y (255)	labd-7,13*E*-dienyl diphosphate	AKP96361	Zerbe et al., 2015
**NtCPS2**	*Nicotiana tabacum*	F (267)	copal-8-ol diphosphate	G3CCC0	Sallaud et al., 2012
**SsLPS**	*Salvia sclarea*	Y (258)	copal-8-ol diphosphate	AET21247	Schalk et al., 2012
**MvCPS1**	*Marrubium vulgare*	Y (256)	peregrinol diphosphate	KJ584450	Zerbe et al., 2014
**MvCPS3**	*M. vulgare*	F (256)	copalyl diphosphate	KJ584452	Zerbe et al., 2014
**CfTPS1**	*Coleus forskohlii*	F (262)	copalyl diphosphate	KF444506	Pateraki et al., 2014
**CfTPS2**	*C. forskohlii*	Y (259)	copal-8-ol diphosphate	KF444507	Pateraki et al., 2014
**SmCPS1**	*S. miltiorrhiza*	F (256)	copalyl diphosphate	AHJ59321	Gao et al., 2009
**SmCPS2**	*S. miltiorrhiza*	F (259)	copalyl diphosphate	AHJ59322	Cui et al., 2015
**TwTPS14**	*Trypterigium wilfordii*	Y (265)	clerodienyl diphoshate	KP889111	Andersen-Ranberg et al., 2016

Supplementary Figures:

```
SdCPSL4       ------------------------------------------------------------
SdCPSL3       ------------------------------------------------------------
SdCPSL2       ------------------------------------------------------------
SdCPSL234     ------------------------------------------------------------
OsCPS4        MPV---FTASFQCVTLFGQPASAADA---Q---------------P--LLQGQRPFLHLH
NtCPS2        MQVIITSSHRFFCHHLH-QLKSPTS---LSAQKAEFKKHGPRNWLF--QTEGSLLY----
CcCLS         ------MAFTFTSAHLF-LPVTEN-----HSVHVNY-SIPPGNWRLWSTAKGG-----SN
Pscps         -------MFTHFSTHFH-LPSS-SSLFFLHPF---Y-KS------S---SLGAVSFVAKD
Atcps         -----------MSLQYH-VLNSIPSTTFLSSTKTTI-SS------SFLTISGSPLNVARD

SdCPSL4       ------------------------------------------------------------
SdCPSL3       --------------------------------------SHIRI--RFVTSNLDLGLFVKV
SdCPSL2       ------------------------------------------------------------
SdCPSL234     --------------------------------------SHIRI--RFVTSNLDLGLFVKV
OsCPS4        --ARRRRPCGPMLISKSPPYPAS----------EETREWEAEGQHEHTDE-------L
NtCPS2        --KPVRLNCA---TSDA-SYLGN------VN--------EYL-ESDHSKNSEEKDIQVSR
CcCLS         KLDIRRLRCS---ARRTPEPLAQGSNGGRDGVEAIQRLQTIA---DDKIDGGANELGIVV
Pscps         KEK-RCRAIS---KSRTQEYEGV--FQTNVAT---LKLSEINVEDVIVIDDEEE------
Atcps         KSRSGSIHCS---KLRTQEYINS--QEVQHDLPLIHEWQQLQGEDAPQISVGSN------

SdCPSL4       ------------------------------------------------------------
SdCPSL3       NKQIEDCVEHVKNLLMTSGDRRISLSPYDTSIVALIEDLE---GREAPQFPSCLECVARH
SdCPSL2       ------------------------------------------------------------
SdCPSL234     NKQIEDCVEHVKNLLMTSGDRRISLSPYDTSIVALIEDLE---GREAPQFPSCLECVARH
OsCPS4        RETTTTMIDGIRTALRSIGEGEISISAYDTSLVALLKRLD---GGDGPQFPSTIDWIVQN
NtCPS2        TIQMKGLTEEIKHMLNSMEDGRLNVLAYDTAWVSFIPNTTNNGNDQRPMFPSCLQWIIDN
CcCLS         WDLIRDGVDAVKSMFDSMGDGDISISAYDTAWVALVKDVN---GSGGPQFPSSLQWIVDN
Pscps         QDIRVGLVNKIKSILSSLEDGEITISAYDTAWVALVEDVN---AISTPQFPSSLEWIAKN
Atcps         SNAFKEAVKSVKTILRNLTDGEITISAYDTAWVALIDAG-----DKTPAFPSAVKWIAEN

SdCPSL4       ----------------------------------NCNQ----------------------
SdCPSL3       QKADDSWGDD-FFCIYDRILNTLACVVALKSWKV--------------------------
SdCPSL2       ------------------------------------------------------------
SdCPSL234     QKADDSWGDD-FFCIYDRILNTLACVVALKSWKVNCNQ----------------------
OsCPS4        QLPDGSWGDASFFMMGDRIMSTLACVVALKSWNIHTDKCERGLLFIQENMWRLAHEEED-
NtCPS2        QLSDGSWGEEIVFCIYDRLLNTLVCVIALTLWNTCLHKRNKGVMFIKENLSKLETGEVE-
CcCLS         QLPDGSWGDSEVFSAYDRLLKTLACVVALKSWNIRPDKCQKGLKFFRDNISKLEKENVEA
Pscps         QLQDGSWGDSRLFSAHDRIINTLACVIALRSWNMHSEKCDKGMIFFRENLSKLENENEE-
Atcps         QLSDGSWGDAYLFSYHDRLINTLACVVALRSWNLFPHQCNKGITFFRENIGKLEDENDE-

SdCPSL4       ------------------------------------------------------------
SdCPSL3       ------------------------------------------------------------
SdCPSL2       ------------------------------------------------------------
SdCPSL234     ------------------------------------------------------------
OsCPS4        --WMLVGFEIALPSLLDMAKDLD-LDIPYDEPALKAIYAERERKLAKIPRDVLHAMPTTL
NtCPS2        --NMTSGFELVFPTLLEKAQQLD-IDIPYDAPVLKDIYARREVKLTRIPKDVIHTIPTTV
CcCLS         SAQMLSGFEVVFLSLIEVARRLD-IQIPLHSPVFEDLIARRNLKFAKIPLDLMHNVPTSL
Pscps         --HMPIGFEVAFPSLLEGARGIKPLMCPNDSPILKNIFEKRDEKLTRIPKEIMHKVPTTL
Atcps         --HMPIGFEVAFPSLLEIARGIN-IDVPYDSPVLKDIYAKKELKLTRIPKEIMHKIPTTL

SdCPSL4       ----------------------AMAPFLTSPSSTAYVFMHTKDEKCFDLIQNAVK-----
SdCPSL3       ------------------------------------------------------------
SdCPSL2       ------------------------------------------------------------
SdCPSL234     ----------------------AMAPFLTSPSSTAYVFMHTKDEKCFDLIQNAVK-----
OsCPS4        LHSLEGMVD--LDWEKLLKLRCLDGSFHCSPASTATAFQQTGDQKCFEYLDGIVKKFNGG
NtCPS2        LFSLEGLRD-DLDWQRLLKLQMPDGSFLISPASTAFAFMETNDEKCLAYLQNVVEKSNGG
CcCLS         LNSLEGMTGVELDWEKLLKLQSQDGSFITSPSSTAFALMQTNDTKCLGYLKFVVQKFNGG
Pscps         LHSLEGMSG--LDWKQLLKLQSQDGSFLFSPSSTAFALMQTKDGNCLKYLNNVVKKFNGG
Atcps         LHSLEGMRD--LDWEKLLKLQSQDGSFLFSPSSTAFAFMQTRDSNCLEYLRNAVKRFNGG
```

```
SdCPSL4       ---NYPVDVFARLSAVDRLQRLGISRFSCHDRTF-------LGIE---------------
SdCPSL3       ------------------------------------------------------------
SdCPSL2       ------------------------------------------------------------
SdCPSL234     ---NYPVDVFARLSAVDRLQRLGISRFSCHDRTF-------LGIE---------------
OsCPS4        VPCIYPLDVYERLWAVDRLTRLGISRHFTSEIED-CLDYIFRNWTP-DGLAHTKNCPVKD
NtCPS2        A-RQYPFDLVTRLWAIDRLQRLGISYYFAEEFKE-LLNHVFRYWDEENGIFSGRNSNVSD
CcCLS         APGQYPVEIFERIWVVDRLQRLGISRYFQLEIKECCLDYAFKHWTQ-YGSSWARNTPVYD
Pscps         VPNVYPVDLFEHIWVVDRLERLGISRFFRHEIKD-CMNYVSKIWSE-KGICWARNSNVQD
Atcps         VPNVFPVDLFEHIWIVDRLQRLGISRYFEEEIKE-CLDYVHRYWTD-NGICWARCSHVQD

SdCPSL4       --KPG-------------------------------------------------------
SdCPSL3       ------------------------------------------------------------
SdCPSL2       -----MDR------------KVFYRLS--SQKKCFFSF----------------------
SdCPSL234     --KPGMDR------------KVFYRLS--SQKKCFFSF----------------------
OsCPS4        IDDTAMGFRLLRLYGYQVDPCVLKKFEKDGKFFCLHGESNPSSVTPMYNTYRASQLKFPG
NtCPS2        VDDTCMAIRLLRLHGYDVSPDALNNFKDGDQFVCFRGEVDG-SPTHMFNLYRCSQVLFPG
CcCLS         LDDTCMAFRILRLHGYDVSAEAFRHFEKNGVFFCFGWETTQ-SVTVNFNLYRATQVAFPG
Pscps         IDDTAMAFRLLRLHGHQVSAHVFKHFERNGEFFCFAGQCTQ-AVTGMYNLFRASQVLFPG
Atcps         IDDTAMAFRLLRQHGYQVSADVFKNFEKEGEFFCFVGQSNQ-AVTGMFNLYRASQLAFPR

SdCPSL4       ------------------------------------------------------------
SdCPSL3       ------------------------------------------------------------
SdCPSL2       -------------------------------------------------SKPVIQY----
SdCPSL234     -------------------------------------------------SKPVIQY----
OsCPS4        DDGVLGRAEVFCRSFLQDRRGSNRMKDKWAIAKDIPGEVEYAMDYPWKASLPRIETRLYL
NtCPS2        EK-ILEEAKNFTYNFLQQCLANNRCLDKWVIAKDIPGEIWYALEFPWYASLPRVEARYYI
CcCLS         EN-ILKEAKQFSFNFLMKKQAAREFQDKWVILKDFPGELKYALEFPWYASLPRVETRFYV
Pscps         EK-ILEHAKHFSAKVLKEKREANELIDKWIIMKNLPEEVGYALDMPWYANLDRIETRFYI
Atcps         EE-ILKNAKEFSYNYLLEKREREELIDKWIIMKDLPGEIGFALEIPWYASLPRVETRFYI

SdCPSL4       ------------------------------------------------------------
SdCPSL3       ------------------------------------------------------------
SdCPSL2       -----------RTYTYMMPEISNDRYKELAILDFNRCQAQHQLKWTYMQEWFHKSSVSEF
SdCPSL234     -----------RTYTYMMPEISNDRYKELAILDFNRCQAQHQLKWTYMQEWFHKSSVSEF
OsCPS4        DQYGGSGDVWIGKVLHRMTLFCNDLYLKAAKADFSNFQKECRVELNGLRRWYLRSNLERF
NtCPS2        EQYGGADDIWIGKTLYRMPDVNNNVYLQAAKLDYNRCQSQHRFEWLIMQEWFEKCNFQQF
CcCLS         EQYGGDNDVWIGKTLYRMPYINNNVYLELAKLDFNNCQALHRKEWETMQKWFMESKLDEF
Pscps         DQYGAESDVWIGKTLYRMAYVNNNNYLELAKLDYNNCQAQHLIEWNVIQTWYLESRLGEF
Atcps         DQYGGENDVWIGKTLYRMPYVNNNGYLELAKQDYNNCQAQHQLEWDIFQKWYEENRLSEW

SdCPSL4       ------------------------------------------------------------
SdCPSL3       ------------------------------------------------------------
SdCPSL2       SIGK--RDLLRAFFLAAATIFEPERTLERLVWTKTQIVSRMITSFLNHGTALSLDQKSLV
SdCPSL234     SIGK--RDLLRAFFLAAATIFEPERTLERLVWTKTQIVSRMITSFLNHGTALSLDQKSLV
OsCPS4        GGTDPQTTLMTSYFLASANIFEPNRAAERLGWARVALLADAVSSHFRRIGGPKNLTSN--
NtCPS2        GISK--KYLLVSYFLAAASIFEVEKSRERLAWAKSRIICKMITSYYNDEATTWTTRNSLL
CcCLS         GVSS--KTLLESYFLAAASIFEPERSTERLAWAKTAFLMETIGSYFDDEMNSKDLRKAFV
Pscps         GLSK--RDLLLAYFLATGSIFEPERSHERLAWAKTTALLETIKCY-VRN---EDLRKDFA
Atcps         GVRR--SELLECYYLAAATIFESERSHERMVWAKSSVLVKAISSSFGES---SDSRRSFS

SdCPSL4       ------------------------------------------------------------
SdCPSL3       ------------------------------------------------------------
SdCPSL2       TQIGHNFDDLDEII-----------------------GYIIL------------------
SdCPSL234     TQIGHNFDDLDEII-----------------------GYIIL------------------
OsCPS4        ---------------------------------------LEELIS------LVPFDDAYS
NtCPS2        MEFKVSHDPTRK--------------NGNETKEILVLKNLRQFLRQLSEETFEDLGKDIH
CcCLS         QEFKNIYE-----------RRMEA--KG--TKWN-LIIILLTTLNH----LTEVCGRDIN
Pscps         KKFNDHIDVRDYSI----ARRM----KRNKTEHE-LVESLFATIGEISWDVRLSYGHEIG
Atcps         DQFHEYIANARRSDHHFNDRNMRLDRPGSVQASR-LAGVLIGTLNQMSFDLFMSHGRDVN
```

```
SdCPSL4       ------------------------------------------------------------
SdCPSL3       ------------------------------------------------------------
SdCPSL2       ------------------------------------------------------------
SdCPSL234     ------------------------------------------------------------
OsCPS4        GSLREAWKQWLMAWTAKESSQESIEGDTAILLVRAIEIFGGRHVLTGQRPDLWEYSQLEQ
NtCPS2        HQLQNAWETWLVFLREEKNACQ----EETELLVRTINLSGGYMTHDE-ILFDADYENLSN
CcCLS         SYLCHSWEKWMMMWEPEGDRYK----GAAELLSNSINLSSGRLFSND-TLSHPNYEKLVT
Pscps         YDMHQCWKKWLSSWQSEGDKCE----GEAELLIQIINLCSNHWISEG-PSMQSTIQHLLQ
Atcps         NLLYLSWGDWMEKWKLYGDEGE----GELM--VKMIILMKNNDLTNF--FTHTHFVRLAE

SdCPSL4       ------------------------------------------------------------
SdCPSL3       ------------------------------------------------------------
SdCPSL2       ------------------------------------------------------------
SdCPSL234     ------------------------------------------------------------
OsCPS4        LTSSICRKLYRRVLAQENGKSTEKVEEIDQQLDLEMQELTRRVLQGCS-AINRLTRETFL
NtCPS2        LTNKVCGKLNELQNDKVTG------GSKNTNIELDMQALVKLVFGNTSSNINQDIKQTFF
CcCLS         LSNKLCHQLGNSRRGNHNE----DSDIKDTKIEIAMQELVQLVHQNSSDDISMDLKQTFF
Pscps         LTNSICHKLSCYQKDKELKGISCQENITNSEVESKMQELVQMVFQKCPNDIDFNVKNTFF
Atcps         IINRICLPRQYLKARRND-----EKEKTIKSMEKEMGKMVELALS--ESDTFRDVSITFL

SdCPSL4       ------------------------------
SdCPSL3       ------------------------------
SdCPSL2       ------------------------------
SdCPSL234     ------------------------------
OsCPS4        HVVKSFCYVAYCSPETIDNHIDKVIFQDVI
NtCPS2        AVVKTFYYSAHVSEEIMNFHISKVLFQQV-
CcCLS         AVVRSFYYAAHCDRGTINSHIVKVLFESVV
Pscps         TIAKSFYYAAFCDSRTINFHIAKVLFEKVV
Atcps         DVAKAFYYFALCGDH-LQTHISKVLFQKV-
```

Supplementary Fig. S1. Sequence alignment of SdCPSL2-4, SdCPSL234 and homologous CPSLs in supplementary Table S5.

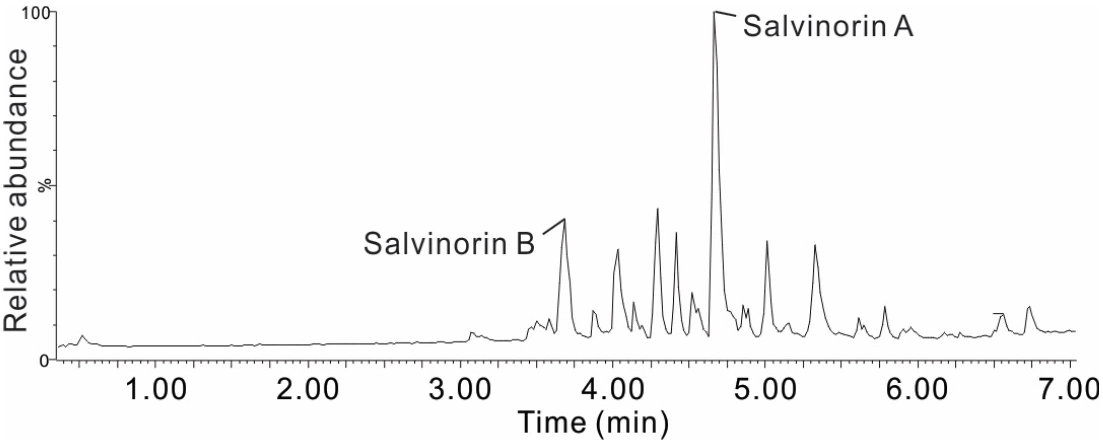

Supplementary Fig. S2. Total ion chromatogram (TIC) from LC-MS analysis under positive ionization mode of peltate trichome metabolite extract.

```
SdKPS   1 ------MSFATSLPRPTTTGAAGFGLPLATCISLSVSHSFSPKF---------------GICNN
Iecps2  1 ---MSSSSNVTSLPRLTTAGGVFPREMVRVHSSCNILRSKAKVG---------------GINYF
Pscps   1 ----MFTHFSTHFHLP-SSSSLFFLHPFYKSSSLGAVSFVAKDK----EK------RCRAISKS
CmCPS2  1 --MSSSSSLSLSRHCL-SSSFSFRLPNLFPPPAPGGCSLRVKDKGAVLESSIRCIIKCNAISKP
Atcps   1 --------MSLQYHVLNSIPSTTFLSSTKTTISSSFLTISGSPLNVARDKSRSGSIHCSKLRTQ
Lscps   1 MKTMISSPIPAFHPRFSPAAGSRR-LSPILP-SSGSVVLTGSKT------------QCKAVSKS
Srcps   1 MKTGFISPATVFHHRISPATTFRHHLSPATTNSTGIVALRDINF------------RCKAVSK-

SdKPS  44 TSLRLKSKAGSGCYEGIHRSQLAASTILEGHTPINPEVESEKIR-LIERIRLMFRSMDDGEISV
Iecps2 47 NPGNIKCVEVHKSRQ--VAVAAVKSLEYETEKPTNQDVVSEKMRVLSERIGTMLQNMNEGEISI
Pscps  50 RTQEYEGVFQTN-VATLKLSEINVEDVIVIDDEEEQDI-RVGLVN---KIKSILSSLEDGEITI
CmCPS2 62 PTQDYSDVLQSG-VPLLKWQQF-VEEGIESETAAQVSV-WEEIEERVKWIKSMLSSMDDGDISI
Atcps  57 EYINSQEVQHDL-PLIHEWQQLQGEDAPQISVGSNSNA-FKEAVK---SVKTILRNLTDGEITI
Lscps  51 PTQEYFDVLQKNGLPFINWQNDVVEDELDKEK-KILYP-NDEIKGFVERIKVMLGSMDEGEITV
Srcps  53 ---EYSDLLQKDEASFTKWDDDKVKDHLDTN--KNLYP-NDEIKEFVESVKAMFGSMNDGEINV

SdKPS  107SPYDTAWVALVEDIG--GSGGPQFPTSLEWISNNQLDDGSWGD-RKFVLYDRILNTLACVVALT
Iecps2 109SPYDTAWVALVEDTD--GR--PQFPTSLEWISNNQLADGSWGD-RKFVIYDRILNTLACVVALT
Pscps  109SAYDTAWVALVEDVN--AISTPQFPSSLEWIAKNQLQDGSWGDSRLFSAHDRIINTLACVIALR
CmCPS2 123SAYDTAWVALIPKVTEEGVKSPQFPSSLEWIANNQLPDGSWGDAQIFSPHDRIINTLASLVALK
Atcps  116SAYDTAWVALIDAGD----KTPAFPSAVKWIAENQLSDGSWGDAYLFSYHDRLINTLACVVALR
Lscps  113SAYDTAWVALVQDID--GNGRPEFPSSLEWIVKNQLSDGSWGDHLIFSAHDRIINTLACVIALT
Srcps  110SAYDTAWVALVQDVD--GSGSPQFPSSLEWIANNQLSDGSWGDHLLFSAHDRIINTLACVIALT

                                           *
SdKPS  168TWKMHPNKCEKGLRFISDNIEKLADEDEELMPVGFEIALPSLIDLAKRLC-IEIPD-NSASIKN
Iecps2 168TWNMHPHKCNRGLRFIRDNIEKLENENEELMPIGFEVVFPSLIEAAQKLG-IEIPHIDSPCIKK
Pscps  171SWNMHSEKCDKGMIFFRENLSKLENENEEHMPIGFEVAFPSLLEGARGIKPLMCPN-DSPILKN
CmCPS2 187SWNLHPQNTRKGVAFFNQNIWKLEEENAEHMPIGFEIAFPSLLEFAKKLK-LGIRS-DSPALQQ
Atcps  176SWNLFPHQCNKGITFFRENIGKLEDENDEHMPIGFEVAFPSLLEIARGIN-IDVPY-DSPVLKD
Lscps  175SWNVHPGKCQKGLKFLNDNISKLEEENPEHMPIGFEVAFPSLIDIARKLD-IQVPE-DSPALKE
Srcps  172SWNVHPSKCEKGLNFLRENICKLEDENAEHMPIGFEVTFPSLIDIAKKLN-IEVPE-DTPALKE

                                    *
SdKPS  232IYAKRDSKLKRIPMDLMHKKPTSLLFSLEGMEGLNWDKLLDFQS-EGSFLSSPSSTAYALHHTK
Iecps2 232IQAMRDFKLKRIPMELLHKKPTSLLHSLEGMQGLVWEKLLDFRS-DGSFLCSPSSTAYALQHTK
Pscps  235IFEKRDEKLTRIPKEIMHKVPTTLLHSLEGMSGLDWKQLLKLQSQDGSFLFSPSSTAFALMQTK
CmCPS2 251INARRRLKLARIPKDIMHKLPTTLLHSLEGMSGLDWEKLLKLQSQDGSFLSSPASTAFALMHTN
Atcps  240IYAKKELKLTRIPKEIMHKIPTTLLHSLEGMRDLDWEKLLKLQSQDGSFLFSPSSTAFAFMQTR
Lscps  239IYARRNLKLTKIPKSLMHKVPTTLLHSLEGMPDLEWEKLLKLQCKDGSFLFSPSSTAFALMQTK
Srcps  236IYARRDIKLTKIPMEVLHKVPTTLLHSLEGMPDLEWEKLLKLQCKDGSFLFSPSSTAFALMQTK

                                *
SdKPS  296DELCLEYLLKAVKKFNGGVPNAYPVDMFEHLWSVDRLRRLGISRYFQVEIDECLDYVYRYWTNK
Iecps2 297DELCLQYLLKAVKKFNGGVPNVYPVDMFEHLWCVDRLQRLGICRYFRAQIKEMLDYVYKYWTDK
Pscps  300DGNCLKYLNNVVKKFNGGVPNVYPVDLFEHIWVVDRLERLGISRFFRHEIKDCMNYVSKIWSEK
CmCPS2 314HPNCFKYLEASVHRFNGGVPNVYPVDLFEHIWAVDRLQRLGISRFFHPHIVECVNNVRTHWSEK
Atcps  304DSNCLEYLRNAVKRFNGGVPNVFPVDLFEHIWIVDRLQRLGISRYFEEEIKECLDYVHRYWTDN
Lscps  303DQKCLQYLTDAVTKFNGGVPNVYPVDLFEHIWVVDRLQRLGISRYFDSEIKDCVDYIYRYWTKD
Srcps  300DEKCLQYLTNIVTKFNGGVPNVYPVDLFEHIWVVDRLQRLGIARYFKSEIKDCVEYINKYWTKN

                       *  **                            *
SdKPS  357GICWARNMCVQDSDDSSMGFRLLRLYGYDVSIDVFKQFEEGGQFCSIPGQMTHAITGMYNLYRA
Iecps2 356GICWARNTNVQDVDDTAMGFRLLRMHGYDVSTDVFKQFEKAGEFCCFPGQSTHAITGMYNVYRT
Pscps  362GICWARNSNVQDIDDTAMAFRLLRLHGHQVSAHVFKHFERNGEFFCFAGQCTQAVTGMYNLFRA
CmCPS2 377GICWARNSEFRDIDDTAMGFRLLRLYGHDVSAEVFKHFEKDGEFVCIAGQSTQAVTGMLNLYRA
Atcps  366GICWARCSHVQDIDDTAMAFRLLRQHGYQVSADVFKNFEKEGEFFCFVGQSNQAVTGMFNLYRA
Lscps  365GICWAKNSNVQDIDDTAMGFRVLRMHGYKVTTDVFRQFEKDGKFVCFPGQTTQAVTGMFNLFRA
Srcps  362GICWARNTHVQDIDDTAMGFRVLRAHGYDVTPDVFRQFEKDGKFVCFAGQSTQAVTGMFNVYRA
```

```
SdKPS  421S-QLMFPQEHILADARNFTANLLHQKRVTNSIVDKWIITKDLPGEVAYALDVPFYASLPR
Iecps2 422S-QIMFDGEDILADAKNYSATFLHQKRLASELVDKWIITKDLPGEVGYALDVPFFASLPR
Pscps  426S-QVLFPGEKILEHAKHFSAKVLKEKREANELIDKWIIMKNLPEEVGYALDMPWYANLDR
CmCPS2 441SDQVMFPGEKILEDAKQFASKFLRQKQAANQLLDKWIIAKDLPGEVGYALDVPWFASLPR
Atcps  430S-QLAFPREEILKNAKEFSYNYLLEKREREELIDKWIIMKDLPGEIGFALEIPWYASLPR
Lscps  429S-QVLFPDEKILEDAKKFSYNYLKEKQSTNELLDKWIIAKDLPGEVEYALDVPWYASLPR
Srcps  426S-QMLFPGERILEDAKKFSYNYLKEKQSTNELLDKWIIAKDLPGEVGYALDIPWYASLPR

SdKPS  480LEARFFLEQYGGDDDVWIGKTLYRMLYVNCNTYLELAKLDYKHCQTVHQLEWNSMQTWYR
Iecps2 481LEARFFLEQYGGDDDVWIGKTLYRMPYVNSDTYLELAKLDYKKCQAVHQLEWKSIQKWYR
Pscps  485IETRFYIDQYGAESDVWIGKTLYRMAYVNNNNYLELAKLDYNNCQAQHLIEWNVIQTWYL
CmCPS2 501VETRLYIQHYGGKNDVWIGKTLYRMFKVNNDTYLELAKLDYNNCQRLHQIEWVDIQKWYT
Atcps  489VETRFYIDQYGGENDVWIGKTLYRMPYVNNNGYLELAKQDYNNCQAQHQLEWDIFQKWYE
Lscps  488LETRFYLEQYGGEDDVWIGKTLYRMGNVSNNTYLEMAKLDYNNCLAIHHLEWNTMQQWYV
Srcps  485LETRYYLEQYGGEDDVWIGKTLYRMGYVSNNTYLEMAKLDYNNYVAVLQLEWYTIQQWYV

SdKPS  540ECNLGEFGLS-ERSLLLAYYIAASTAFEPEKSSERLAWAITTILVETIMSQELS----DE
Iecps2 541DCKLGEFGLG-EKRLLLAYFLAASTAFEPEKKGERLAWAKTAFLVETIASQQLS----HE
Pscps  545ESRLGEFGLS-KRDLLLAYFLATGSIFEPERSHERLAWAKTTALLETIKCYVR----NED
CmCPS2 561ESKLRDYGMR-RSSILFSYFGAVCSIFEPERAKERLAWTKTAALVHTIASHYKDA--NAH
Atcps  549ENRLSEWGVR-RSELLECYYLAAATIFESERSHERMVWAKSSVLVKAISSSFGE---SSD
Lscps  548DFGMERFGTSDITSLLVSYYLAAASVFEPERSKERIAWAKTTTLVDTISSFFHSLKISNE
Srcps  545DIGIEKFESDNIKSVLVSYYLAAASIFEPERSKERIAWAKTTILVDKITSIFDSSQSSKE

SdKPS  599QKREFVDEFVNISI------------INNQNGGRYKPGNRLVEVLINTV------TLMAE
Iecps2 600QKREFPNEFEHGSS------------LNMENGGRYKTRTRLVEILSNTVSQLSFETLVAE
Pscps  604LRKDFAKKFNDHID-----VRDYS-IARRMK--RNKTEHELVESLFATIGEISWDVRLSY
CmCPS2 620QRRAFLQQFTNFHA-----AQPYDNNAWRSGNMQQKGGEGLVGILLRTLTNISLDILLSH
Atcps  608SRRSFSDQFHEYIANARRSDHHFNDRNMRLDRPGSVQASRLAGVLIGTLNQMSFDLFMSH
Lscps  608HRREFVEEFRNISN-------------SIHHAKYGKPWHGLMVALKGTLHEIALDVLMTH
Srcps  605DITAFIDKFRNKSS-------------SKKHSINGEPWHEVMVALKKTLHGFALDALMTH

SdKPS  643GRGTDQQLSNAWKNWLKTWEEGGDLGEAEARLLLHTIHLSSG--LDESSFSHPKYQQLLE
Iecps2 644GRDIKQQLSNTWQKWLKTWEEGGNLGEAEAQLLLQTLHLSSG--LDESSFSHPKYHQLLE
Pscps  653GHEIGYDMHQCWKKWLSSWQSEGDKCEGEAELLIQIINLCSNHWISEGPSMQSTIQHLLQ
CmCPS2 673GVDITHHLHQAWQKWVFKWQEDGDVHKEEAELLVQTIILNSGCSTLEDLLSNSQFQKLSN
Atcps  665GRDVNNLLYLSWGDWMEKWKLYGD--EGEGELMVKMIILMKNNDLTN-FFTHTHFVRLAE
Lscps  655RRDIHPQLHHAWEMWLMRWQQGVDATEGQAELIVQTINMTAGRWVSNELLAHPQYRLLSS
Srcps  652SQDIHPQLHQAWEMWLTKLQDGVDVT---AELMVQMINMTAGRWVSKELLTHPQYQRLST

SdKPS  697ATSKVCHQLRLFQNLKANDAQGSTSRLVTVTTFQIEAGMQELVKLIFTKTL--EDLTSAT
Iecps2 704ATCKVCNQLRLFQNRKAHDAQGGISDLVIGTTFQIEASMQELVKLVFTKSS--EDLDSIT
Pscps  712LTNSICHKLSCYQKDKELKGISCQENITN---SEVESKMQELVQMVFQKCP--NDIDFNV
CmCPS2 733LTNKVCHQLAHFKKHKVNNGNLYKEKTDNKMPPEIEEDIRKLVQLVIQKSSDGNDIDSPI
Atcps  723IINRICLPRQYLKARRNDE----KEKTIK---SMEKEMGKMVELALSESD----TFRDV-
Lscps  715VINNICHEIYHNRT--------CMEVNSTTISTSIDSKMQELVQLVLSDSL--DDLDQDL
Srcps  709VTNSVCHDITKLHN--------FKEN-----STTVDSKVQELVQLVFSDTP--DDLDQDM

SdKPS  755KQSFFNIARSFYYTAYCPADTIDSHINKVLFEKIV
Iecps2 762KQSFFAIARSFYYTAYCDAGAINSHIYKVLFENID
Pscps  769KNTFFTIAKSFYYAAFCDSRTINFHIAKVLFEKVV
CmCPS2 793KQTFLTVAKSVYYAAYFDAWTINYHIAKVLFERVF
Atcps  775SITFLDVAKAFYYFALCG-DHLQTHISKVLFQKV-
Lscps  767KQTFLTVAKTFYYKAYCDPETINVHISKVMFETII
Srcps  756KQTFLTVMKTFYYKAWCDPNTINDHISKVFEIVI-
```

Supplementary Fig. S3. Sequence alignment of SdKPS and CPSs as indicated in the results. Asterisks indicate residues mutated in this study. The conserved DXDD motif is underlined. The predicted N-terminal transit peptide of SdKPS that is removed for recombinant expression in this study is highlighted in yellow.

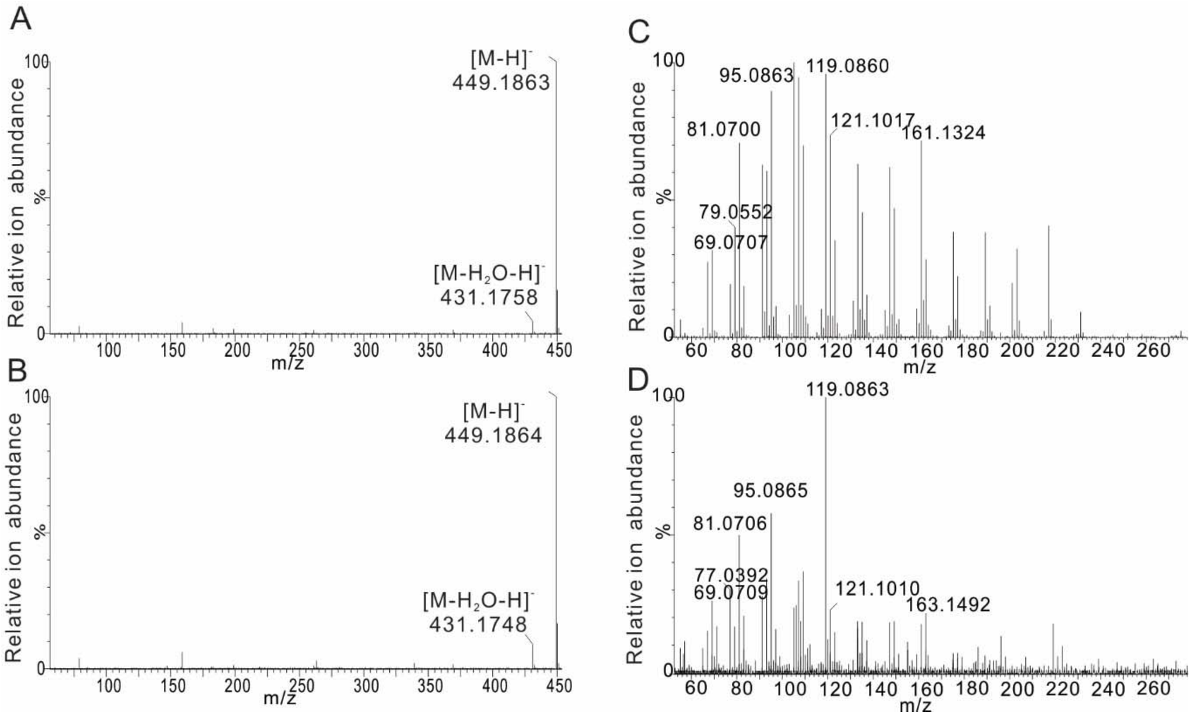

Supplementary Fig. S4. LC-MS/MS spectra of peaks in Fig. 5. A, peak a (449.1863 *m/z*, [M-H]$^-$). B, peak b (449.1864 *m/z*, [M-H]$^-$). C, peak d (273.2581 *m/z*, [M-H_2O+H]$^+$). D, peak e (273.2581 *m/z*, [M-H_2O+H]$^+$).

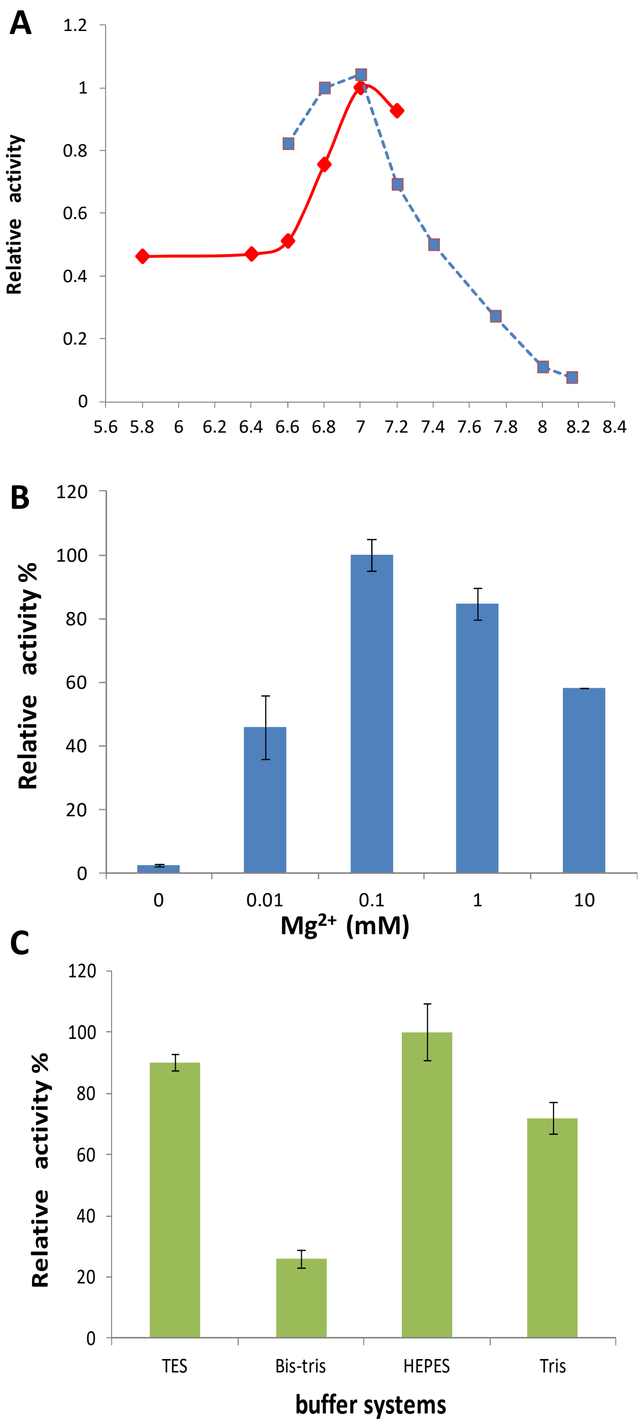

Supplementary Fig. S5. Determination of optimal conditions for *in vitro* enzymatic reaction of SdKPS. Dependence of reaction rates on (A) pH, (B) concentration of Mg^{2+}, and (C) buffer systems. Values are shown as mean ± SE from three biological replicates.

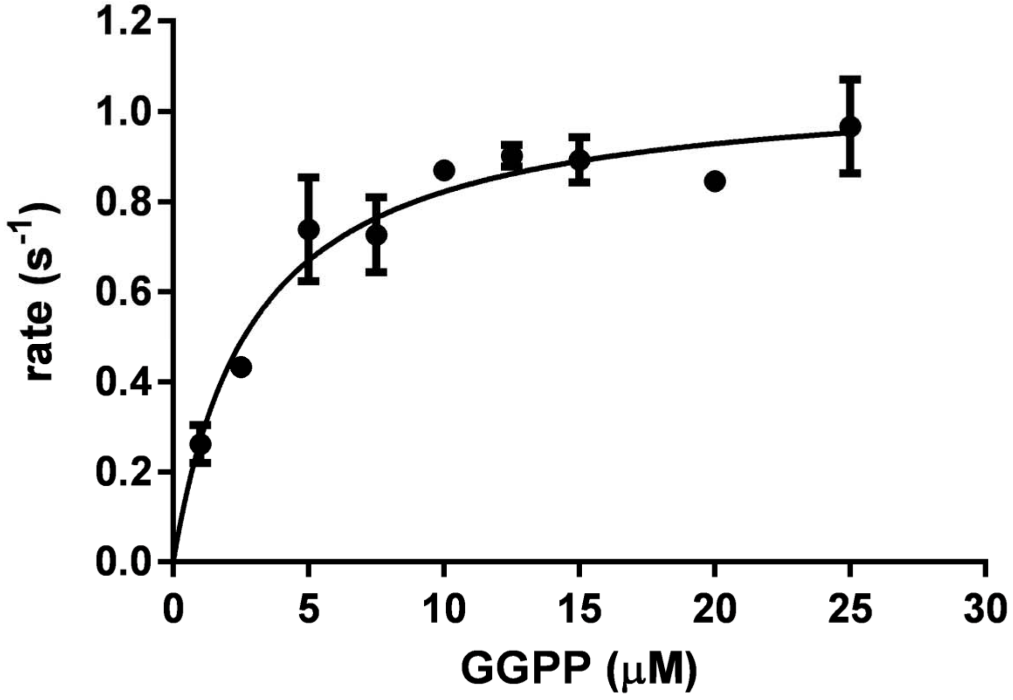

Supplementary Fig. S6. A representative hyperbolic plot of SdKPS reaction rate vs. substrate concentration. Assays were repeated 3 times and values of K_M and *kcat* were calculated as mean ± SE from replicates.

A

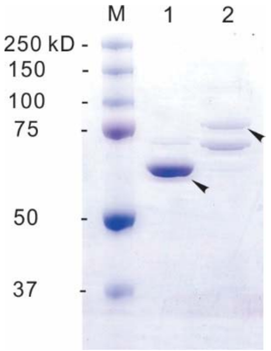

B

SdKSL1: MSLAFNAEVM AFSVNRVREV FPVR

SdKSL3: MSLPLSTCLL LSPSESHFRR FRFSTASASL KAGLQ

Supplementary Fig. S7. SDS-PAGE of purified recombinant SdKSL1 and SdKSL3 and their putative N-terminal transit peptide sequences. A, SDS-PAGE of purified truncated proteins. M= standard. 1: SdKSL1 (Molecular Weight = 68 KD). 2: SdKSL3 (Molecular Weight =87 KD). Arrows indicate the target protein band. B, putative N-terminal transit peptide sequences of SdKSL1 and SdKSL3.

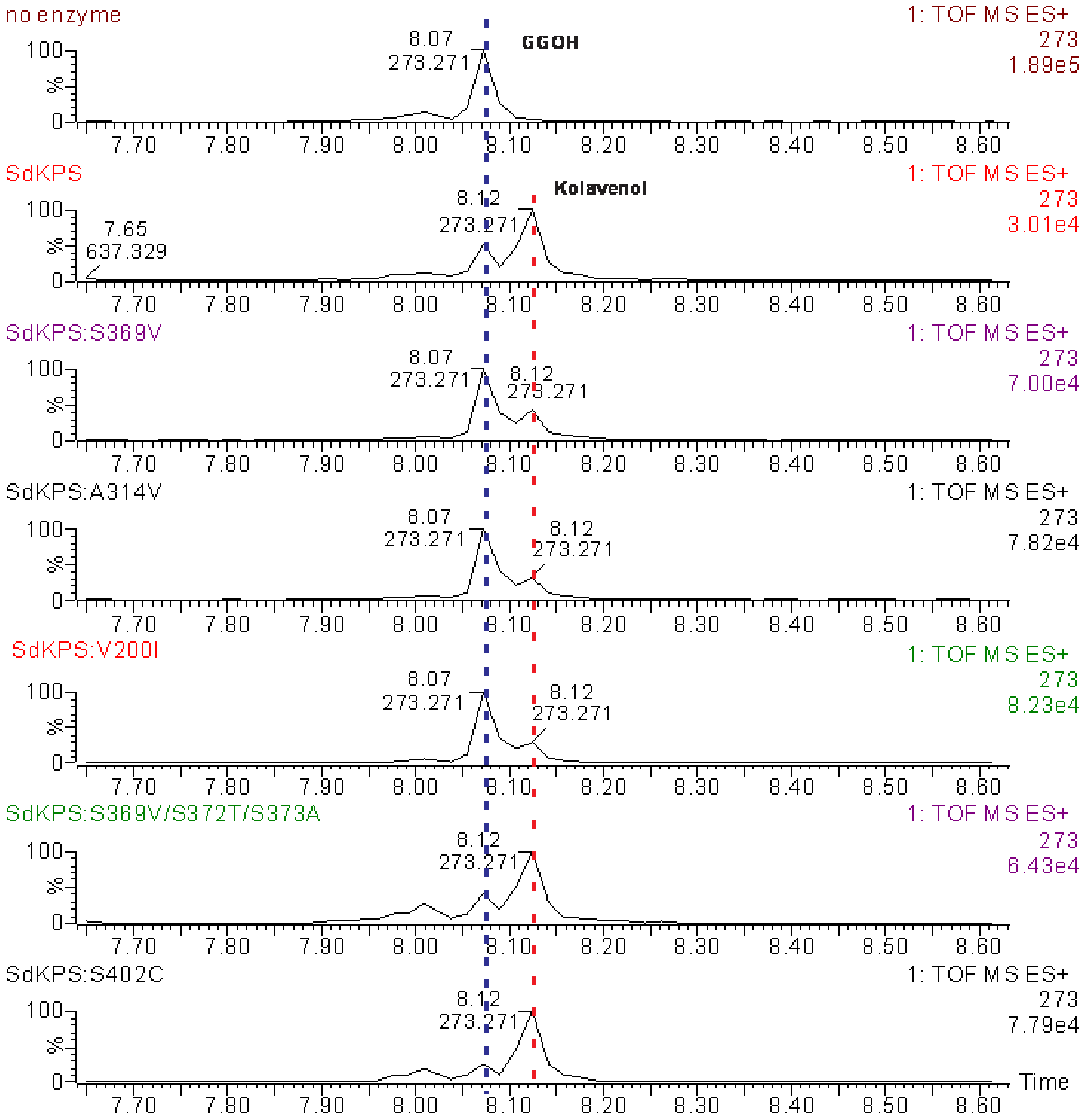

Supplementary Fig. S8. LC-MS extracted ion chromatograms (*m/z* 273) of products of SdKPS and other mutants with GGPP. Enzymes are indicated on the upper left corner of each chromatogram.
